# The Possible Earliest Allopolyploidization in Tracheophytes Revealed by Phylotranscriptomics and Morphology of Selaginellaceae

**DOI:** 10.1093/molbev/msae153

**Published:** 2024-08-05

**Authors:** Jong-Soo Kang, Ji-Gao Yu, Qiao-Ping Xiang, Xian-Chun Zhang

**Affiliations:** State Key Laboratory of Plant Diversity and Specialty Crops, Institute of Botany, Chinese Academy of Sciences, Beijing 100093, China; State Key Laboratory of Plant Diversity and Specialty Crops, Institute of Botany, Chinese Academy of Sciences, Beijing 100093, China; University of Chinese Academy of Sciences, Beijing 100049, China; China National Botanical Garden, Beijing 100093, China; State Key Laboratory of Plant Diversity and Specialty Crops, Institute of Botany, Chinese Academy of Sciences, Beijing 100093, China; China National Botanical Garden, Beijing 100093, China; State Key Laboratory of Plant Diversity and Specialty Crops, Institute of Botany, Chinese Academy of Sciences, Beijing 100093, China; China National Botanical Garden, Beijing 100093, China

**Keywords:** *Selaginella sanguinolenta*, Selaginellaceae, lycophytes, phylotranscriptomics, ancient hybridization, nuclear phylogeny, allopolyploidization, whole-genome duplication (WGD)

## Abstract

Selaginellaceae, originated in the Carboniferous and survived the Permian–Triassic mass extinction, is the largest family of lycophyte, which is sister to other tracheophytes. It stands out from tracheophytes by exhibiting extraordinary habitat diversity and lacking polyploidization. The organelle genome-based phylogenies confirmed the monophyly of *Selaginella*, with six or seven subgenera grouped into two superclades, but the phylogenetic positions of the enigmatic *Selaginella sanguinolenta* clade remained problematic. Here, we conducted a phylogenomic study on Selaginellaceae utilizing large-scale nuclear gene data from RNA-seq to elucidate the phylogeny and explore the causes of the phylogenetic incongruence of the *S*. *sanguinolenta* clade. Our phylogenetic analyses resolved three different positions of the *S*. *sanguinolenta* clade, which were supported by the sorted three nuclear gene sets, respectively. The results from the gene flow test, species network inference, and plastome-based phylogeny congruently suggested a probable hybrid origin of the *S*. *sanguinolenta* clade involving each common ancestor of the two superclades in Selaginellaceae. The hybrid hypothesis is corroborated by the evidence from rhizophore morphology and spore micromorphology. The chromosome observation and Ks distributions further suggested hybridization accompanied by polyploidization. Divergence time estimation based on independent datasets from nuclear gene sets and plastid genome data congruently inferred that allopolyploidization occurred in the Early Triassic. To our best knowledge, the allopolyploidization in the Mesozoic reported here represents the earliest record of tracheophytes. Our study revealed a unique triad of phylogenetic positions for a hybrid-originated group with comprehensive evidence and proposed a hypothesis for retaining both parental alleles through gene conversion.

## Introduction

Lycophyte shares a common ancestor with the other tracheophyte, dating back nearly 400 million years, making it a key group to study the origin and evolution of land plants (Banks et al. 2011). Selaginellaceae is the largest family of lycophytes, consisting of a single genus, *Selaginella*, with approximately 750 species and a cosmopolitan distribution (Jermy 1990; Zhang et al. 2013). *Selaginella* has attracted broad attention for its distinctive genome evolution, such as lacking whole-genome duplication (WGD) events, unconventional organelle genomes, and environmental adaptation through desiccation-tolerant species (Banks et al. 2011; Jiao et al. 2011; Li et al. 2015; Baniaga et al. 2016; VanBuren et al. 2018; Xu et al. 2018; Mower et al. 2019; Zhang et al. 2019a, 2019b; Kang et al. 2020; Xiang et al. 2022). Plants of *Selaginella* are herbaceous and heterosporous. There is extensive morphological variation in the position of rhizophores, the shapes of vegetative leaves and sporophylls, leaf arrangements, and sculptures of mega- and microspores, as well as different basic chromosome numbers and ploidy levels among species (Zhou and Zhang 2015; Weststrand and Korall 2016a).

The phylogenetic incongruence has emerged as an interesting scientific question in the Selaginellaceae originating from the Carboniferous period, particularly related to the *Selaginella sanguinolenta* clade (Korall and Kenrick 2004; Weststrand and Korall 2016b; Zhang et al. 2020; Tang et al. 2023). According to the latest infrageneric classification, *Selaginella* consists of seven subgenera: *Selaginella*, *Rupestrae*, *Lepidophyllae*, *Gymnogynum*, *Exaltatae*, *Ericetorum*, and *Stachygynandrum* (Weststrand and Korall 2016a). The *S. sanguinolenta* clade morphologically belongs to subg. *Stachygynandrum* and currently includes seven species, such as *S. sanguinolenta*, *S. rossii*, *S. nummularifolia*, *S. aitchisonii*, *S. jacquemontii*, *S. sajanensis*, and a recently described species, *S. baodongii* (Zhang et al. 2023). However, the phylogenetic position of the *S. sanguinolenta* clade remains unresolved across different gene trees. This clade formed a sister relationship with all-rhizophoric *Selaginella* in phylogenies using the plastid *rbcL*, nuclear ribosomal ITS region, and a nuclear single copy gene (Zhou et al. 2015a; Weststrand and Korall 2016b), but appeared as sister to only subg. *Stachygynandrum* in another nuclear single copy gene tree (Weststrand and Korall 2016b).

Phylogenetic incongruence persists even when analyzing data from two different organelle genomes, plastid genome (plastome) and mitochondrial genome (mitogenome). While plastome-based phylogenomic studies place the *S. sanguinolenta* clade as sister to subg. *Stachygynandrum* (Zhang et al. 2020; Zhou et al. 2022), the mitogenome-based phylogenomic study reveals a sister relationship with all the rhizophoric subgenera, except the non-rhizophore subg. *Selaginella* (Tang et al. 2023). Phylogenetic incongruence between different gene trees can result from different evolutionary processes, including gene duplication and loss, hybridization, gene transfer and introgression, incomplete lineage sorting (ILS), and evolutionary rate variation (Maddison 1997). However, it is challenging to disentangle these alternatives in scenarios, especially when the events happened long ago (Stull et al. 2023). Nonetheless, recent advancements in data acquisition and analytical tools have made distinguishing among alternative hypotheses possible (Goulet et al. 2017; Folk et al. 2018; Kubatko and Chifman 2019; Wang et al. 2020a; Stull et al. 2023).

Transcriptome sequencing (RNA-seq) has been demonstrated as an efficient approach to generate large-scale nuclear gene data for phylogenomic study (Hittinger et al. 2010; Wen et al. 2013; Huang et al. 2016a, 2016b), allowing for the resolution of deep phylogenetic relationships of major clades (Wickett et al. 2014; Zeng et al. 2014; Huang et al. 2016a, 2016b; Xiang et al. 2017; Qi et al. 2018; Ran et al. 2018; Shen et al. 2018; Zhang et al. 2022; Chen et al. 2023). Here, we conducted a comprehensive phylogenomic study of *Selaginella* using RNA-seq data. This study aimed to provide a perspective on species relationships from the nuclear genome data by constructing a detailed nuclear data-based phylogeny, evaluate the various phylogenetic positions of the *S. sanguinolenta* clade, and explore the evolutionary processes underlying the observed diversification pattern in this early diverged and evolutionary key family of tracheophytes.

## Results

### Phylogenetic Reconstruction of *Selaginella*

**Fig. 1.**
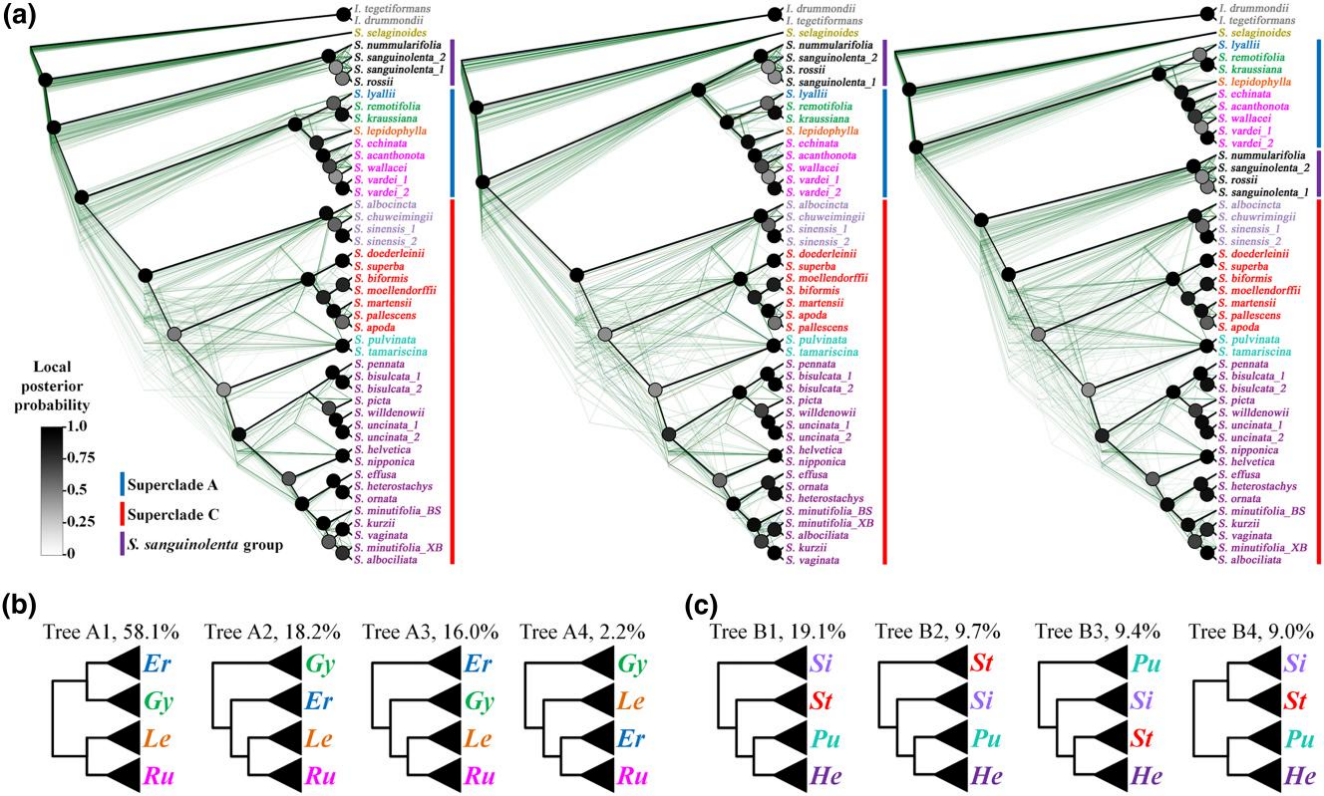
Coalescent-based nuclear phylogenies and topology frequencies for each group in Selaginellaceae. a) The coalescent-based species trees (black line) and single gene trees (green lines) for 130, 81, and 136 gene sets, showing different positions of the *S. sanguinolenta* clade. The node color represents local posterior support values calculated by ASTRAL, according to the scale shown on the left. Bootstrap support values from concatenated ML analyses of the three gene sets were all strongly supported at over 90% (supplementary fig. S1, Supplementary Material online). Taxa marked in different colors represent each subgenus or clade within superclades A and C. Vertical bars marked in blue, purple, and red represent superclades A, the *S. sanguinolenta* clade, and superclade C, respectively. Three groups, *Isoetes* (outgroup), subg. *Selaginella*, and the *S. sanguinolenta* clade not included in superclades A and C, are indicated in gray, yellow, and black, respectively. b) The most common topologies among single gene trees within superclade A, with their frequencies indicated. *Er*, subg. *Ericetorum*; *Gy*, subg. *Gymnogynum*; *Le*, sug. *Lepidophyllae*; *Ru*, subg. *Rupestrae*. c) The most common topologies among single gene trees within superclade C, with their frequencies indicated. *Si*, *S. sinensis* clade; *St*, subg. *Stachygynandrum* sensu Zhou and Zhang (2015); *Pu*, *S. pulvinata* clade; *He*, subg. *Heterostachys* sensu Zhou and Zhang (2015).

**Fig. 2.**
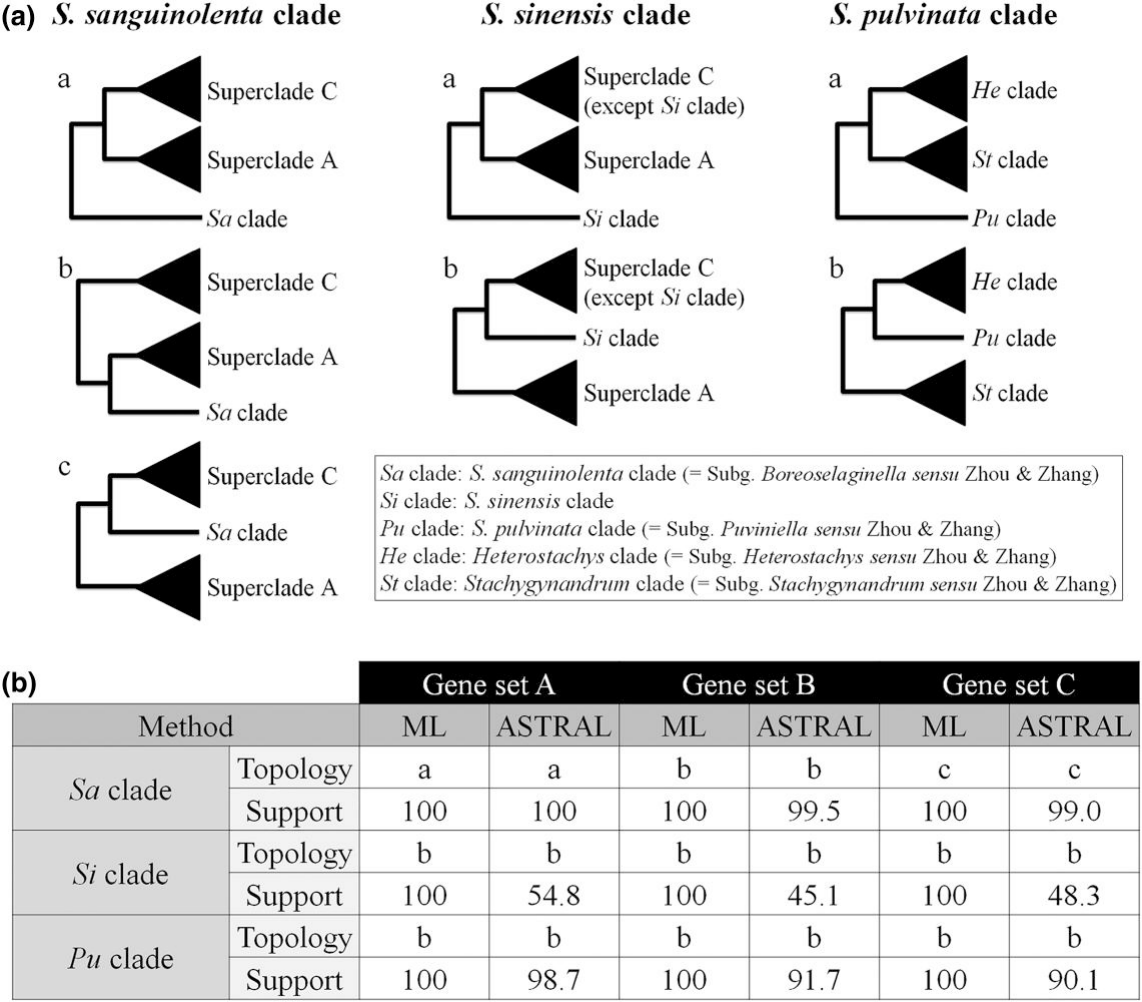
Summary of different phylogenetic relationships of the three enigmatic clades in Selaginellaceae. a) Simplified topologies showing different phylogenetic relationships of three different clades. For the *S. sanguinolenta* (*Sa*) clade, the three topologies are obtained in this study, and the “topology b” is a newly found one in this study. For the *S. sinensis* (*Si*) clade and the *S. pulvinata* (*Pu*) clade, the “topology a” is obtained using plastid genome data from a previous study (Zhou et al. 2022), and the “topology b” represents the most frequent topology among the 347 single/low copy nuclear gene trees obtained in this study. b) Bootstrap support values and local posterior probabilities of each topology from concatenated ML and coalescent methods. Superclade A, the *S. sanguinolenta* clade, and superclade C are marked as blue, purple, and red in Fig. 1a, respectively.

More than 3,000 putative single/low copy nuclear gene groups were used to select the orthologous genes. Considering that all accessions possess at least one transcript and examining whether each group is monophyletic, we used 13 representative species covering the phylogenetic diversity in this family for the first round of paralog removal, resulting in 1,025 putative orthologous genes chosen for the second step. The second removal incorporated two criteria from the first removal and selected those putative orthologous genes with an aligned length of over 1,000 bp, applying this to all 46 accessions. A total of 347 orthologous genes were eventually obtained for further phylogenetic analyses. According to the results of the substitution saturation test conducted by DAMBE, all 347 orthologous genes used for phylogenetic reconstruction in this study were confirmed to be unsaturated (supplementary table S1, Supplementary Material online). While confirming the monophyly for each group, we found that the *S. sanguinolenta* clade was located at three different positions, with two positions reported previously (Zhou et al. 2015a; Weststrand and Korall 2016b) and one newly found in this study (Fig. 1a). Based on the well-supported positions of the *S. sanguinolenta* clade, we categorized the selected orthologous genes into three gene sets (Fig. 1a). The three different phylogenetic positions of the *S. sanguinolenta* clade are as follows: gene set A, comprising 130 orthologous genes, the *S. sanguinolenta* clade is sister to all-rhizophoric *Selaginella* composed of superclades A and C; gene set B, comprising 81 orthologous genes, the clade is sister to superclade A, which includes the subgenera *Ericetorum*, *Gymnogynum*, *Lepidophyllae*, and *Rupestrae*; gene set C, comprising 136 orthologous genes, the clade is sister to superclade C, which includes subg. *Stachygynandrum* (Figs. 1a and2a). The first and third positions of the *S. sanguinolenta* clade, inferred from gene sets A and C, respectively, were previously reported (Zhou and Zhang 2015; Weststrand and Korall 2016b; Du et al. 2020; Zhang et al. 2020; Chen et al. 2022; Tang et al. 2023). However, the position inferred from gene set B was newly revealed in this study (Fig. 1a). The three positions inferred from three different gene sets were strongly supported by both concatenated ML and coalescent ASTRAL methods, respectively (Figs. 1a and 2b, supplementary fig. S1, Supplementary Material online). Upon investigating the functional roles of genes within each gene set, it was observed that their differing topologies appear unrelated to their functions. Information on the 347 orthologous genes ultimately selected for this study is provided in supplementary table S2, Supplementary Material online.

Except for the position of the *S. sanguinolenta* clade, the phylogenetic results of the three gene sets showed a consistent topology inferred from concatenated ML and coalescent ASTRAL methods with strong support values (Fig. 1a, supplementary fig. S1, Supplementary Material online). Although the monophyly of subgenus *Selaginella*, subg. *Ericetorum*, and subg. *Lepidophyllae* cannot be confirmed as only one species from each was included, the monophyly of each subgenus, subg. *Gymnogynum*, subg. *Rupestrae*, and subg. *Stachygynandrum* sensu Weststrand and Korall, was strongly supported in all phylogenetic analyses (Fig. 1a). *Selaginella selaginoides*, representing subg. *Selaginella* characterized by lacking the rhizophore, was resolved as being the sister of the remainder of the genus, a clade that produces rhizophores, with strong support (Fig. 1a). The rhizophoric clade contains three major clades, named superclades A, B, and C for convenience. Superclade A is composed of four subgenera, *Rupestrae*, *Lepidophyllae*, *Gymnogynum*, and *Ericetorum*, and superclade C is composed of subg. *Stachygynandrum* sensu Weststrand and Korall (2016a) (Fig. 1a). Superclade B consists of *S. sanguinolenta*, *S. rossii*, and *S. nummularifolia* in subg. *Stachygynandrum* sensu Weststrand and Korall (2016a). Within superclade A, both concatenated ML and coalescent ASTRAL analyses indicated that subgenera *Ericetorum* and *Gymnogynum* formed a subclade, and subgenera *Lepidophyllae* and *Rupestrae* formed the other subclade (Fig. 1a). This topology was the most frequent among the 347 orthologous genes (Fig. 1b). Within superclade C, analyses using both methods placed the *S*. *sinensis* clade as the sister of the remaining groups of the superclade, and the *S. pulvinata* clade (=subg. *Pulviniella* sensu Zhou and Zhang) was sister to the *Heterostachys* clade (=subg. *Heterostachys* sensu Zhou and Zhang) (Fig. 1a). This topology was the most frequent in the 347 single gene trees (close to 20% frequency), and three other topologies were close to 10% each (Fig. 1c). Four of the five sections in the *Heterostachys* clade, according to Zhou and Zhang (2015), were included in this study. Section *Homostachys* (*S. nipponica* and *S. helvetica*) and sect. *Oligomacrosporangiatae* (*S. uncinata*, *S. willdenowii*, *S. picta*, *S. bisulcata*, and *S. pennata*) were each monophyletic with strong supports, and together, they formed a monophyletic group, whereas sect. *Heterostachys* (*S. kurzii*) was nested within sect. *Tetragonostachyae* (Fig. 1a). In the *Stachygynandrum* clade (=subg. *Stachygynandrum* sensu Zhou and Zhang), five of seven sections were included in this study. Section *Ascendentes* (*S. doederleinii* and *S. superba*) and sect. *Pallescentes* (*S. pallescens* and *S. apoda*) formed their own clades; however, the monophyly of the other three sections could not be evaluated due to the single sampling (Fig. 1).

### Assessments of Allopolyploidization Origin of the *S. sanguinolenta* Clade

We used the Ks distribution to further explore the evolutionary histories of the *S. sanguinolenta* clade, including *S. sanguinolenta*, *S. nummularifolia*, and *S. rossii*. By comparing representatives from two superclades putatively involved in an allopolyploidization event that gave rise to the clade, we calculated the Ks for each gene of *S. vardei* (subg. *Rupestrae* in superclade A) and *S. sinensis* (subg. *Stachygynandrum* in superclade C) based on phylogenetic results. The Ks values are then plotted according to their frequencies to observe their distribution. We constructed the Ks distributions of all duplicated gene pairs (paralogs) for each of the three species within the *S. sanguinolenta* clade, as well as for *S. vardei* and *S. sinensis* (supplementary fig. S2, Supplementary Material online; supplementary table S3, Supplementary Material online). Transcriptome data often include multiple transcripts from the same gene, such as alternatively spliced transcripts and false paralogs (during assembly), which have very high similarity (Ren et al. 2018; Wang et al. 2019). These can affect the Ks distribution similarly to recently duplicated genes, making it difficult to detect ancient genome duplications (Cui et al. 2006; Ren et al. 2018; Wang et al. 2019; Zwaenepoel et al. 2019). Therefore, we constructed Ks distributions before and after filtering gene pairs with very high similarity (90% or higher) to identify signals of ancient genome duplications based on the cutoff Ks distribution (supplementary fig. S2, Supplementary Material online).

Comparison of the Ks distributions before and after applying the cutoff indicated that the cutoff primarily affected the peak at Ks < 0.5 (supplementary fig. S2b and c, Supplementary Material online). After removing these highly similar sequences, the peak near Ks = 1.5 in the *S. sanguinolenta* clade is significantly enhanced, while those in *S. sinensis* and *S. vardei* were diminished (supplementary fig. S2c, Supplementary Material online). This suggests that the apparent peak in *S. sinensis* and *S. vardei* may be due to unfiltered, highly similar transcripts. Thus, highly similar transcripts can obscure the inference of ancient polyploidization events, emphasizing the necessity and effectiveness of the cutoff method in refining Ks distribution analysis (Cui et al. 2006; Ren et al. 2018; Wang et al. 2019). The cutoff Ks distributions of duplicated gene pairs within each transcriptome revealed two significant Ks peaks (around 1.5 and 3.0) in all three species of the *S. sanguinolenta* clade (supplementary fig. S2, Supplementary Material online). In contrast, the *S. vardei* and *S. sinensis* transcriptomes only showed a large peak at Ks values above 3.0. While a possible paleo-polyploidization event (around 3.0) was shared by the five representative species as inferred by a previous study (Wang et al. 2020b), the recent polyploidization event (around 1.5) is presumed to be independent only in the *S. sanguinolenta* clade and not shared with the species of superclades A and C.

**Fig. 3.**
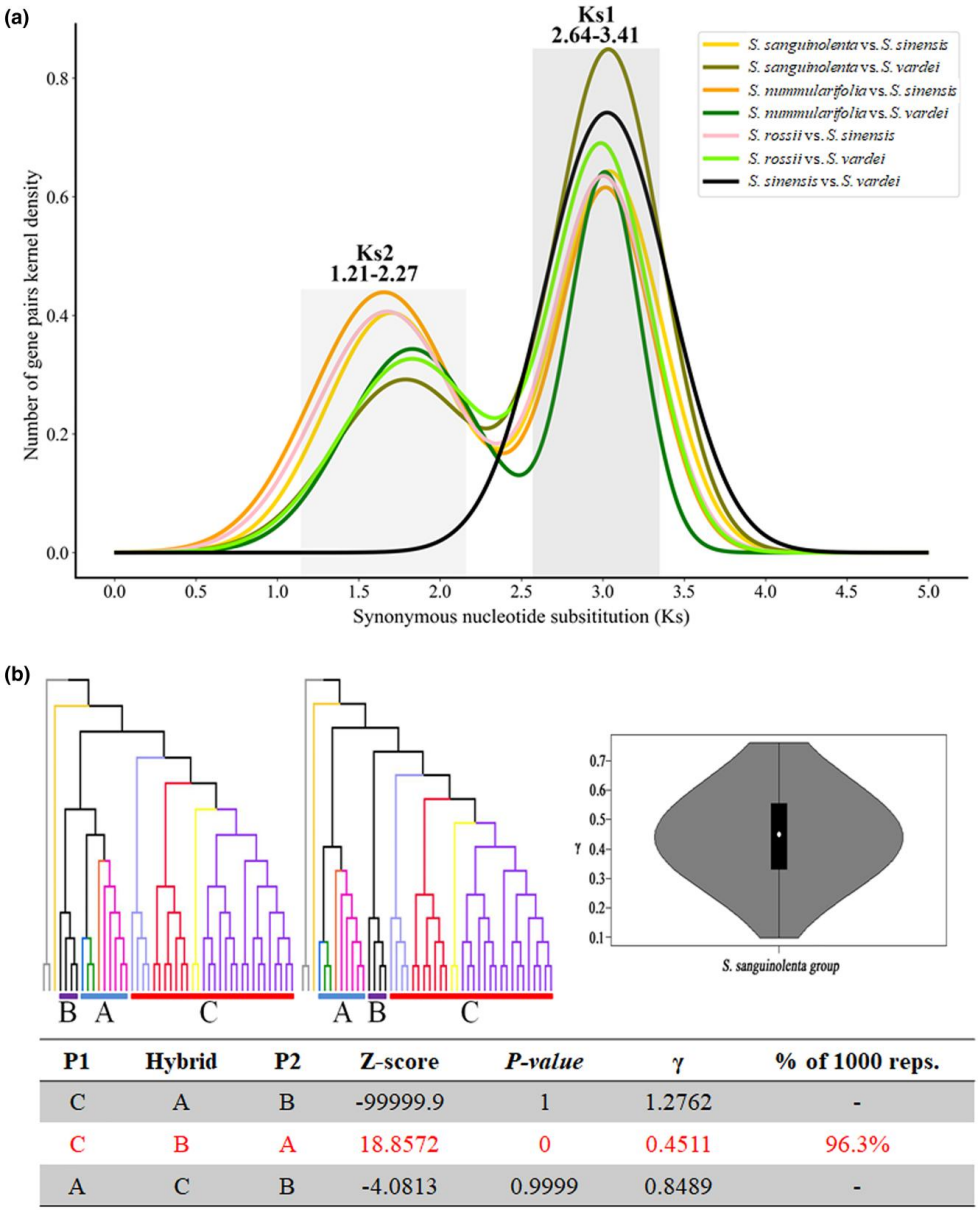
Evidence of allopolyploidization from distribution of synonymous substitution and intergroup gene flow. a) The number of synonymous substitutions per synonymous sites (Ks) distributions confirming the occurrence of an allopolyploidization event in the *S. sanguinolenta* clade (*S. sanguinolenta*, *S. nummularifolia*, and *S. rossii*). The different curves indicate the normal distribution of Ks values in Ks1 (dark gray box) and Ks2 (gray box) regions. b) The results of intergroup gene flow test using HyDe. Designated three groups for the gene flow test. Superclade A (including four subgenera), the *S. sanguinolenta* clade, and superclade C (subg. *Stachygynandrum*) are marked as groups A, B, and C, respectively. Violin plot of distribution of γ calculated by the HyDe across 1,000 bootstrap replicates. γ and 1-γ indicate inheritance probabilities from the putative parental groups A and C, respectively. Test results of detecting hybridization among the three groups are shown in the table.

We constructed the interspecific Ks distributions by applying the same cutoff as used for the Ks distribution of paralogous genes within each species (supplementary fig. S3, Supplementary Material online). Our cutoff interspecific Ks distributions obviously revealed a single peak, Ks1, between *S. vardei* and *S. sinensis* near 3.03 (Fig. 3a, supplementary fig. S3, Supplementary Material online; supplementary table S4, Supplementary Material online), representing the genetic differentiation after the divergence of the two superclades A and C. In contrast, two peaks were detected when comparing the three species of the *S. sanguinolenta* clade with *S. vardei* or *S. sinensis*, respectively. One of these peaks (3.00 to 3.04) aligns closely with Ks1, which can be explained by inferring the genetic differentiation between the *S. sinensis* component in the *S. sanguinolenta* clade and *S. vardei*, or the *S. vardei* component in the *S. sanguinolenta* clade and *S. sinensis*. The other peak, the younger Ks2, ranged from 1.21 to 2.27, representing genetic differentiation between the *S. sinensis* component in the *S. sanguinolenta* clade and *S. sinensis* (1.21 to 2.11), and that between the *S. vardei* component in the *S. sanguinolenta* clade and *S. vardei* (1.34 to 2.27). The presence of the Ks2 peak specifically when comparing the *S. sanguinolenta* clade and each superclade is congruent with the allopolyploidy hypothesis, possessing genomic components from both superclades A and C. The Ks2 peak is shared by all species of the *S. sanguinolenta* clade.

Furthermore, to determine whether the 347 orthologous genes used in the phylogenetic reconstruction were related to genes that were duplicated through allopolyploidization, we performed a Ks distribution using these 347 genes (supplementary fig. S4, Supplementary Material online; supplementary table S5, Supplementary Material online). As a result, a Ks peak was formed around 1.5 between the *S. sanguinolenta* clade and *S. sinensis*, and a Ks peak was formed around 1.9 between the *S. sanguinolenta* clade and *S. vardei*, confirming that the genes used in the phylogenetic reconstruction were related to the Ks2 peak (1.21 to 2.27) of the interspecific Ks distribution (Fig. 3a). Consequently, when considering the results of Ks distributions between species and within a genome, it can be inferred that the species within the *S. sanguinolenta* clade have likely experienced a common allopolyploidization event.

### Intergroup Gene Flow and Species Network Inference

**Fig. 4.**
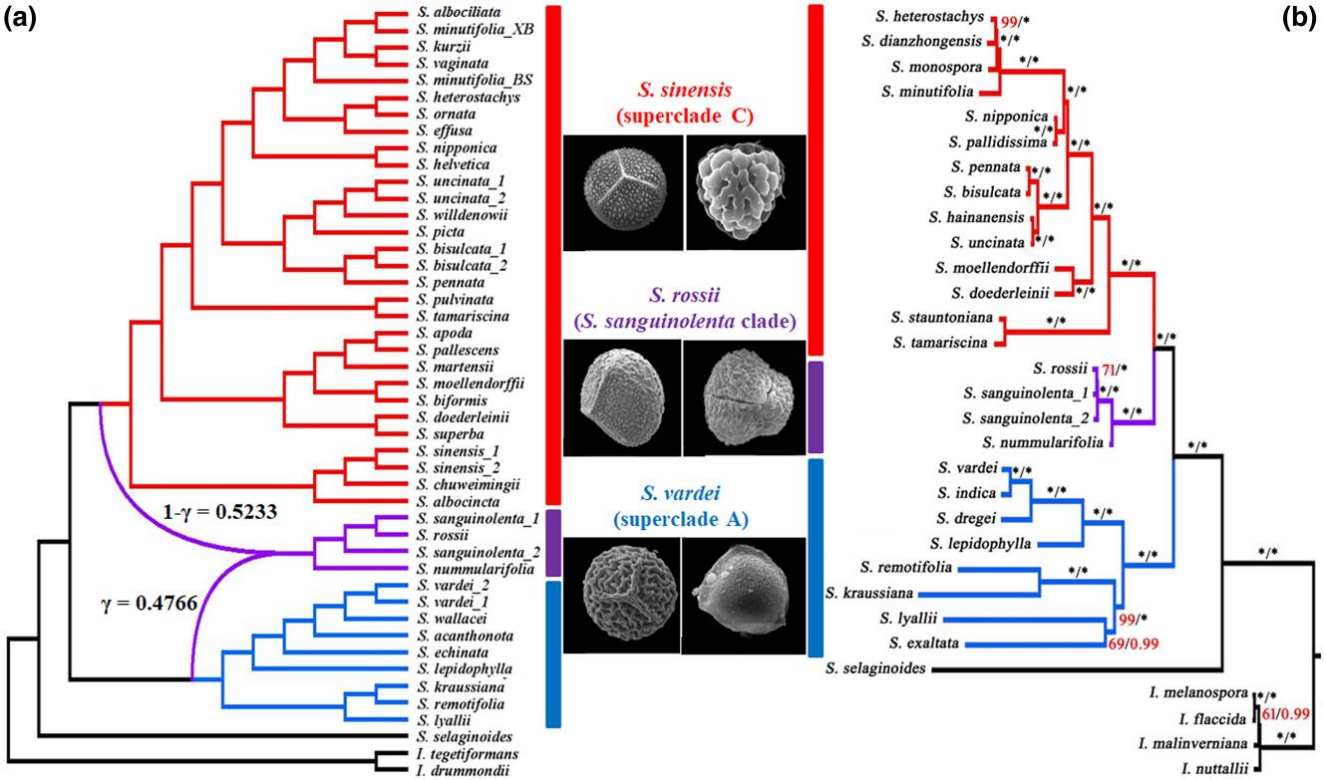
Species network inference from nuclear phylogenies and plastome-based phylogeny of Selaginellaceae. a) Species network inferred from PhyloNet. b) A plasome-based phylogeny adapted from Zhang et al. (2020). The γ and 1-γ values indicate inheritance probabilities from the common ancestors of superclade A (blue) and superclade C (red), respectively. The values near the nodes are bootstrap and posterior probabilities from the ML and BI analyses, respectively. Asterisks indicate 100% supports, whereas the values lower than 100% support are shown in red. The megaspore (left) and microspore (right) of representative species for each clade are presented in the middle of the species network inference a) and the plastome-based phylogeny b).

Since the *S. sanguinolenta* clade was monophyletic and sister to each or both of the two superclades (Fig. 1), we defined three groups in *Selaginella* to test the gene flow and infer the species network. The three clearly defined groups are as follows: (i) group A for superclade A, (ii) group B for the *S. sanguinolenta* clade, and (iii) group C for superclade C (Fig. 3b). The three groups were used as ingroups, and *S. selaginoides* (subg. *Selaginella*) and two *Isoetes* species were defined as outgroups for the HyDe test. A total of 444,491 nucleotides from the three gene sets were used for this gene flow test. The result showed a significant hybridization signal in the *S. sanguinolenta* clade (Z-score = 18.8572, *P* = ∼0.0, γ = 0.4511) (Fig. 3b). Of the 1,000 bootstrap replicates, 96.3% detected hybridization in the *S. sanguinolenta* clade, and the estimated γ values (the inheritance probability from an ancestor of group A in this study) ranged from 0.0991 to 0.6559. The species network inference, using PhyloNet, suggested that the *S. sanguinolenta* clade (group B) was hybrid origin, and the potential parents were the common ancestors of superclades A and C, respectively (Fig. 4a). The estimated inheritance probabilities (γ) indicated that the genetic composition of the *S. sanguinolenta* clade was 47.6% from the common ancestor of superclade A (γ; group A), and the remaining 52.3% from the common ancestor of superclade C (1-γ; group C) (Fig. 4a). These results were consistent with the results from the hybridization detection using HyDe, as well as the inheritance probabilities (Fig. 3b).

### Morphological and Cytological Evidence

To find morphological and chromosomal evidence of the hybridization between the common ancestors of the two superclades, creating the *S. sanguinolenta* clade, morphological and cytological characters were compared among subgenera or groups in *Selaginella* (supplementary table S6, Supplementary Material online). The leaf arrangements evolved from spirally arranged to four-rows of vegetative leaves and sporophylls, and the leaf morphology evolved from monomorphic to dimorphic in shape for vegetative leaves and sporophylls (supplementary table S6, Supplementary Material online). Subgenus *Selaginella*, which is sister to all-rhizophoric *Selaginella*, possesses several ancestral characters, including the helical arrangement of vegetative leaves and sporophylls and the absence of rhizophores (supplementary table S6, Supplementary Material online). The position of the rhizophore is a key character that distinguishes between superclades A and C. The superclade A is characterized by the dorsal rhizophores, whereas the superclade C is characterized by the ventral rhizophores (supplementary table S6, Supplementary Material online). The *S. sanguinolenta* clade has rhizophores on the dorsal side (supplementary fig. S5, Supplementary Material online), which is consistent with the superclade A, although it is morphologically similar to the superclade C in vegetative leaves, sporophylls, and strobili (Zhang et al. 2013).

In spore morphology, the two superclades can be distinguished by the surface sculptures of megaspores. Most species within superclade C, excluding those in the *Stachygynandrum* clade (=subg. *Stachygynandrum* sensu Zhou and Zhang), have been reported to possess megaspores with a verrucate surface, whereas species within superclade A are reported to have a reticulate surface (Korall and Taylor 2006; Zhou et al. 2015b; Vaganov et al. 2019). In this study, we observed the mega- and microspores of *S. vardei*, representing subg. *Rupestrae* (superclade A), *S. sinensis*, representing the *S. sinensis* clade (superclade C), and *S. sanguinolenta*, representing the *S. sanguinolenta* clade (supplementary fig. S6, Supplementary Material online; supplementary table S6, Supplementary Material online). Consistent with the previous observations (Korall and Taylor 2006; Zhou et al. 2015b), the megaspores of *S. vardei* and *S. sinensis* were observed to have reticulate and verrucate surfaces, respectively (supplementary fig. S6, Supplementary Material online; supplementary table S6, Supplementary Material online). The megaspore surfaces of *S. sanguinolenta* were not reticulate like in *S. vardei* or verrucate like in *S. sinensis* (supplementary fig. S6, Supplementary Material online; supplementary table S6, Supplementary Material online). Instead, the megaspores of *S. sanguinolenta* have verrucae that are closely packed together but do not form a reticulate pattern. This surface sculpture appears to represent an intermediate form between the reticulate megaspores observed in *S. vardei* and the verrucate megaspores observed in *S. sinensis*.

Moreover, the chromosome number of the *S. sanguinolenta* clade differed from species in the two superclades. According to previous cytological studies (Jermy et al. 1967; Love and Love 1975, 1976; Zhukova and Petrovsky 1975; Takamiya 1993; Mukhopadhyay and Goswami 1996), species within superclade A typically exhibit a chromosome number of 2n = 18 or 20 (supplementary fig. S6, Supplementary Material online). In superclade C, although variations in chromosome numbers, including a hexaploidy (*S. martensii*) in the *Stachygynandrum* clade and a basic chromosome number reduction (9 to 8) in the *Heterostachys* clade, where *S. heterostachys* shows tetraploidy (2n = 32), were observed, the majority of species in this superclade generally maintain a chromo*some number of 2n = 18 or 20 (supplementary fig. S7, Supplementary Material online). Our chromosome observation of *S. sinensis*, representing the *S. sinensis* clade in superclade C, indicated that the chromosome number of *S. sinensis* was 2n = 18 (supplementary fig. S8, Supplementary Material online). Although 18 or 20 chromosomes are observed in most *Selaginella* species and are thus inferred to be the ancestral chromosome numbers (Jermy et al. 1967; Love and Love 1975, 1976; Zhukova xand Petrovsky 1975; Takamiya 1993; Mukhopadhyay and Goswami 1996), the *S. sanguinolenta* clade was reported to have 30 chromosomes (supplementary fig. S8, Supplementary Material online; supplementary table S6, Supplementary Material online). The 30 chromosomes were consistent in different species of the *S. sanguinolenta* clade, e.g. *S. rossii* collected from Japan (Takamiya 1993) and *S. sanguinolenta* collected from China (this study; supplementary fig. S8, Supplementary Material online), suggesting that the 30 chromosomes are probably the synapomorphy of the whole *S. sanguinolenta* clade.

### Molecular Dating for the Allopolyploidization Event

**Fig. 5.**
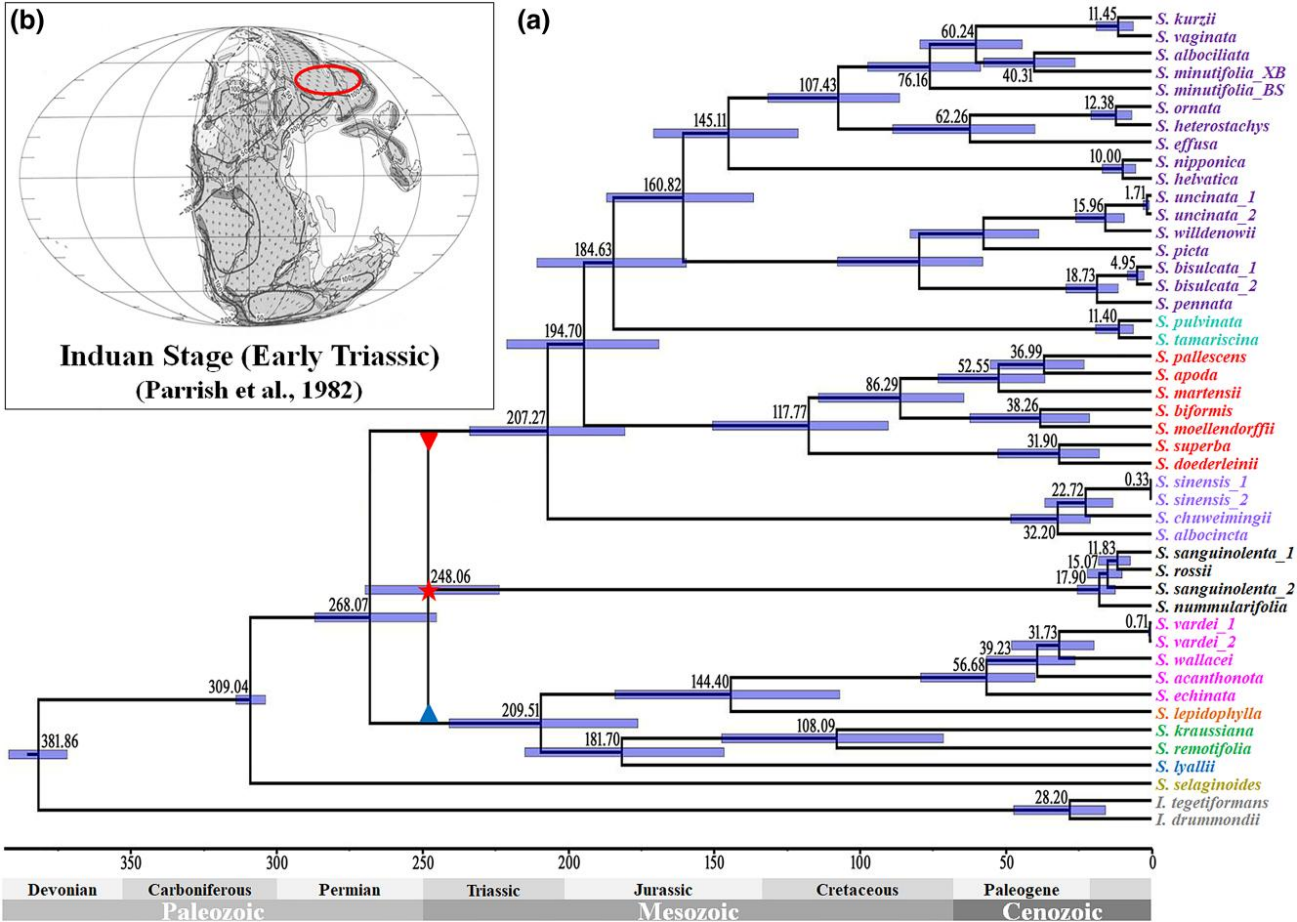
Time estimation of the hybridization between the two superclades in the Selaginellaceae creating the *S. sanguinolenta* clade. a) Divergence times for Selaginellaceae and the estimated time for the ancient hybridization that created the *S. sanguinolenta* clade. The red star indicates the hybridization between the common ancestors of each superclade. The backbone of divergence time was inferred from gene set C using the MCMCtree method, since the divergence times inferred from gene sets B and C were almost consistent. b) The Induan stage in the Earliest Triassic, adopted from Parrish et al. (1982). The red circle indicates the current northern parts of China and some Siberian regions.

Based on the inference that the *S. sanguinolenta* clade originated through the allopolyploidization between each common ancestor of superclades A and C, respectively (Figs. 3b and 4), we used gene sets B and C. These gene sets are expected to be inherited from each parental lineage. We used them to estimate the divergence time of the *S. sanguinolenta* clade, which resolves as a sister to superclades A or C in gene sets B or C, respectively (Figs. 1 and 3b). We believe that this approach would be proper for molecular dating of the allopolyploidization event, which is not bifurcated, as well as minimizing the impact of gene flow in the mixed datasets. Using PL and MCMCtree methods, the estimated divergence times based on two different gene sets (B and C) are shown in supplementary fig. S9, Supplementary Material online. The divergence times inferred by the PL method were slightly older than those by the MCMCtree method (supplementary fig. S9, Supplementary Material online). Comparing the results from the two methods, the estimated times between the two gene sets differed more when applying the PL method (supplementary fig. S9a, Supplementary Material online), whereas the estimations of the two gene sets were almost consistent when using the MCMCtree method (supplementary fig. S9b, Supplementary Material online). Furthermore, a mega-fossil of *Selaginella anasazia* (Ash 1972) found in the Late Triassic was regarded as an ancestral species of subg. *Gymnogynum*, and the minimum age of the fossil was set at 210 Ma (Arrigo et al. 2013). The fossil of *S. anasazia* exhibited dimorphic and decussate vegetative leaves as well as monomorphic sporophylls, aligning well with the two subgenera in this study, *Gymnogynum* and *Ericetorum* (supplementary table S6, Supplementary Material online). However, the divergence times between the clade of the two subgenera and the clade of subgenera *Rupestrae* and *Lepidophyllae* inferred by the PL method (around 170 Ma) were much younger than the fossil's minimum age (210 Ma) (supplementary fig. S9a, Supplementary Material online), and the divergence time estimated by the MCMCtree method for subgenera *Gymnogynum* and *Ericetorum* was 196 and 209 Ma (supplementary fig. S9b, Supplementary Material online), which closely approximates the fossil's minimum age. Therefore, we eventually applied the MCMCtree method for the divergence time estimation (Fig. 5a).

The divergence times of the two different datasets inferred by MCMCtree indicated that the split between superclades A and C occurred at ca. 268 Ma (supplementary fig. S9b, Supplementary Material online; supplementary table S7, Supplementary Material online). The divergence times estimated by gene sets B and C indicated that the split between superclades A and C occurred at 267 Ma (t*_i_* in supplementary fig. S10, Supplementary Material online) and 268 Ma (t*_k_* in supplementary fig. S10, Supplementary Material online), respectively (supplementary fig. S9b, Supplementary Material online). Between two different gene sets, the split time consistency of two putative parental clades (t*_i_* and t*_k_*) implies that allopolyploidization is more likely to cause the incongruence in this family (Sang and Zhong 2000) (supplementary fig. S10, Supplementary Material online). Likewise, the estimated split times between the *S. sanguinolenta* clade and each superclade (A or C) were also consistent, although the topologies were different (Figs. 1 and 4, and supplementary fig. S9, Supplementary Material online). The divergence time between the *S. sanguinolenta* clade and superclade A (t*_h1_* in supplementary fig. S9, Supplementary Material online) was around 247 Ma (268 to 223 Ma in 95% highest posterior density; HPD) in gene set B, and that between the *S. sanguinolenta* clade and superclade C (t*_h2_* in supplementary fig. S10, Supplementary Material online) was around 248 Ma (269 to 223 Ma in 95% HPD) in gene set C (supplementary fig. S9b, Supplementary Material online).

Alternatively, to confirm whether the split time between the *S. sanguinolenta* clade and superclade C (maternal lineage) is consistent with the split time estimated by gene set C, we estimated divergence times for the Selaginellaceae based on maternally inherited plastid genome data (supplementary fig. S11, Supplementary Material online; supplementary table S7, Supplementary Material online). The estimated divergence times using plastid genome data indicated that the split time between two superclades was 272.63 Ma (289 to 248 Ma in 95% HPD), and the split time between the *S. sanguinolenta* clade and superclade C was 245.06 Ma (269 to 215 Ma in 95% HPD), which are consistent with the estimated divergence times based on gene set C (supplementary figs. S9 and S11, Supplementary Material online; supplementary table S7, Supplementary Material online). Given the consistent split time between the *S. sanguinolenta* clade and each superclade in our analyses using gene sets B and C, as well as plastid genome data, the allopolyploidization event yielding the *S. sanguinolenta* clade probably happened at ca. 248 Ma (269 to 215 Ma) during the Early Triassic (Fig. 5a).

## Discussion

### Phylogenetic Relationships Within *Selaginella*

*Selaginella* species display complicated evolutionary patterns with reversals and/or parallelisms in morphology, such as phyllotaxy (spiral vs. decussate) and leaf heteromorphism (isophylly vs. anisophylly) (Korall and Kenrick 2002; Zhou et al. 2015a; Weststrand and Korall 2016b). The phylogenetic relationships within this early-diverged lineage of tracheophytes remain unclear yet. Recently, different relationships among subgenera were reported based on the plastid *rbcL*, 26S nrDNA, and two nuclear single copy genes, *pgiC* and *SQD1* (Korall and Kenrick 2004; Zhou et al. 2015a; Weststrand and Korall 2016b). The conflicting relationships among subgenera have not been settled even in phylogenetic analyses using the plastid and mitochondrial genome sequences (Du et al. 2020; Tang et al. 2023). Three plastome-based and one mitogenome-based studies uncovered the incongruent infrageneric relationships within this family, particularly within superclades A (comprising four subgenera) and C (including subg. *Stachygynandrum*) (Du et al. 2020; Zhang et al. 2020; Zhou et al. 2022; Tang et al. 2023). Our phylotranscriptomic analyses confirmed that subg. *Selaginella*, composed of *S. selaginoides* and *S. deflexa*, characterized by lacking rhizophores, was sister to the remaining species of *Selaginella*, all of which possess rhizophores (Fig. 1a). Considering the morphology of spiral leaf arrangement (in subg. *Selaginella*) and the phylogenetic relationships of the other two families of lycophytes, Lycopodiaceae and Isoetaceae (Chen et al. 2022), both of which exhibit spiral phyllotaxy, the spiral phyllotaxy might be plesiomorphic of Selaginellaceae. Therefore, this relationship aligned with the unique morphological distinction of subg. *Selaginella* from the other subgenera, and its most basal position of the genus (supplementary table S6, Supplementary Material online).

Morphologically, all five subgenera in superclade A are commonly featured by ventral rhizophores and monomorphic and tetrastichous sporophylls (supplementary table S6, Supplementary Material online). The sister relationship between subgenera *Rupestrae* and *Lepidophyllae* was strongly supported by plastome- and mitogenome-based phylogenies (Du et al. 2020; Zhang et al. 2020; Zhou et al. 2022; Tang et al. 2023), but the phylogenetic relationships among subgenera *Gymnogynum*, *Ericetorum*, and *Exatatae* differed depending on data partitioning strategies (Du et al. 2020). Our results confirmed the close relationships between subgenera *Rupestrae* and *Lepidophyllae* (over 90% frequency among total topologies), and between subgenera *Gymnogynum* and *Ericetorum* (the most frequent one) (Fig. 1b), although lacking species of subg. *Exaltatae*. Except for subg. *Selaginella*, subg. *Rupestrae* is the other lineage possessing the spiral phyllotaxy in the family. It is sister to subg. *Lepidophyllae*, which is characterized by decussate phyllotaxy and nearly monomorphic vegetative leaves (supplementary table S6, Supplementary Material online). Likewise, a sister relationship between subg. *Ericetorum*, mostly with monomorphic vegetative leaves, and subg. *Gymnogynum*, with dimorphic vegetative leaves, was the most frequent topology in our phylogenies (Fig. 1a and b). Integrating the results of morphological comparison and phylogenies, the phyllotaxy is inferred to evolve from spiral to decussate and the leaf heteromorphism from monomorphic to dimorphic in both vegetative leaves and sporophylls. The exceptional morphology seen in some species is probably the evolutionary evidence of reverse and/or parallelism, as discussed in previous studies (Korall and Kenrick 2002; Zhou et al. 2015a; Weststrand and Korall 2016b). In addition, we recovered three different topologies with high frequencies in superclade A, where one showing most frequent (58.1%) and the two others with nearly equal frequencies (18.2% and 16.0%) (Fig. 1b). This frequency among three different topologies was previously regarded as a typical signature of the ILS (Pamilo and Nei 1988; Zhang et al. 2019c) (supplementary fig. S10, Supplementary Material online). Therefore, we speculate that the ILS process is probably responsible for the phylogenetic incongruences among subgenera within superclade A.

The phylogenetic relationships within superclade C are more complicated, and the phylogenetic position of the *S. sinensis* clade still remains controversial. The *S. sinensis* plastome exhibited unique genomic and genetic features, including a multipartite genome structure, nucleotide substitution rates elevated two or three times higher compared to other *Selaginella* plastomes, and a GC content of 36.2%, markedly lower than the typical over 50% observed in other *Selaginella* plastomes (Xiang et al. 2022). The *S. sinensis* clade was placed as the sister to all other *Selaginella* species, leading with a long branch, as indicated by the plastome data (Zhou et al. 2022). This sister relationship was determined to be the result of long-branch attraction caused by the extremely accelerated nucleotide substitution rates (Xiang et al. 2022). However, its mitogenome features were unremarkable in the genus. The mitogenome phylogenetic position of the *S. sinensis* clade was nested within superclade C (Tang et al. 2023), and was sister to the clade composed of the *Heterostachys* clade (=subg. *Heterostachys* sensu Zhou and Zhang) and the *S. pulvinata* clade (=subg. *Pulviniella* sensu Zhou and Zhang). Then, the formed clade was sister to the *Stachygynandrum* clade (=subg. *Stachygynandrum* sensu Zhou and Zhang) within superclade C (Fig. 1c). The frequency of the topology inferred from mitogenome-based phylogeny was about 9.7% of the total revealed by transcriptome data in our study. The most frequent (19.1%) position of the *S. sinensis* clade revealed by transcriptome data is the most basal of superclade C (Fig. 1a and c), which is robustly supported by morphological characters, such as ventral rhizophores, dimorphic leaves, decussate phyllotaxy, and verrucate megaspore surfaces (supplementary figs. S5 and S6, Supplementary Material online; supplementary table S6, Supplementary Material online).

The other interesting question is the phylogenetic position of the *S. pulvinata* clade, which was resolved as sister to the clade composed of the *Heterostachys* and *Stachygynandrum* clades by plastid phylogenies (Korall and Kenrick 2004; Zhou et al. 2015a, 2022; Weststrand and Korall 2016b; Du et al. 2020; Zhang et al. 2020; Chen et al. 2022; Zhang and Zhang 2022). However, our transcriptome-based results indicated that the *S. pulvinata* clade and the *Heterostachys* clade formed a monophyletic clade first, and then a sister to the *Stachygynandrum* clade (Figs. 1 and 2). This position was suggested by the plastome-based phylogeny when considering RNA editing events (Du et al. 2020) and the mitogenome-based phylogeny (Tang et al. 2023). According to our previous studies, the *Selaginella* organelle genomes possess pervasive RNA editing to restore the accelerated mutations probably caused by the improper DNA repair system (Kang et al. 2020, 2022; Zhang et al. 2020; Xiang et al. 2022). The consensus position of the *S. pulvinata* clade, achieved by the plastomes considering RNA editing, the mitogenomes, and transcriptomes, demonstrates the power of integrative evidence of different genomes and the deep understanding of the data.

### The *S. sanguinolenta* Clade: The Possible Earliest Allopolyploidization in Tracheophytes

Hybridization associated with polyploidy, also known as allopolyploidization, is an important evolutionary force that can generate biological diversity, especially in plants (Stebbins 1950; Grant 1981; Soltis and Soltis 2009; Goulet et al. 2017; Folk et al. 2018; Stull et al. 2023). Elucidating ancient hybridization events is challenging because the genetic traits inherited from both parental lineages tend to diminish over time due to mutation and recombination (Moody and Rieseberg 2012; Folk et al. 2018). Evolutionary processes, gene duplication and loss, gene transfer and introgression, hybridization, ILS, and evolutionary rate variation, commonly account for phylogenetic incongruence among genes. In our case with *Selaginella*, multiple lines of evidence consistently suggested that hybridization mostly likely accounts for the phylogenetic incongruence of the *S. sanguinolenta* clade, and other processes are less likely, which can be ruled out as discussed below. First, gene duplication and loss were excluded from our dataset in the process of complying with the orthologous gene datasets. Second, if gene transfer and introgression, which differ from hybridization in gene frequency, were responsible for the phylogenetic incongruence, we would observe one predominant topology in the majority of single gene trees, while other topologies were less frequent (Folk et al. 2018). However, in Selaginellaceae, genes associated with each of the three positions of the *S. sanguinolenta* clade are prevalent in the nuclear genome, as evidenced by the counts of 130 (37.46%), 81 (23.34%), and 136 (39.19%) genes out of the 347 single/low copy genes compiled for phylogenetic analysis (Fig. 1a).

**Fig. 6.**
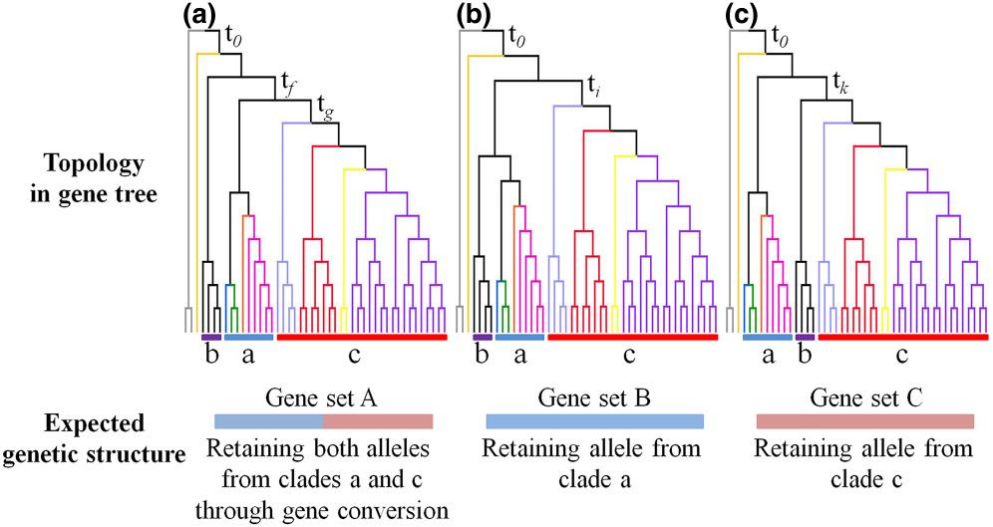
Scheme of the expected selective pressure corresponding to the three position of the *S. sanguinolenta* clade. a, superclade A including four subgenera, b, the *S. sanguinolenta* clade, c, superclade C including subg. *Stachygynandrum* sensu Weststrand and Korall (2016a). t*_0_*, t*_f_*, t*_g_*, t*_i_*, t*_j_*, and t*_k_*, indicate divergence times.

It can be exceptionally challenging to determine whether hybridization or ILS is the cause of phylogenetic incongruence. We explored this challenge by considering both the estimated split times and the frequency distribution of the three different topologies in Selaginellaceae. A significant difference between t*_i_* (split time between superclade A and the clade composed of the *S. sanguinolenta* clade and superclade C) and t*_k_* (split time between superclade C and the clade composed of the *S. sanguinolenta* clade and superclade A) is expected if the conflict topologies were caused by ILS (Sang and Zhong 2000) (supplementary fig. S10, Supplementary Material online). However, the estimated t*_i_* and t*_k_* were very close based on different datasets in this family (Fig. 6). In addition, if ILS caused the conflicting topologies, one topology is expected to be the most frequent (species tree), while the other two less frequent topologies (alternative topologies) would be nearly equal (Pamilo and Nei 1988) (supplementary fig. S10, Supplementary Material online). However, the frequencies of the three topologies observed in this family showed that two of the topologies have similar frequencies; one is obviously the least frequent, not the most frequent. Furthermore, while ILS is usually associated with rapid diversification (Pamilo and Nei 1988; Maddison 1997; Pease et al. 2016; Feng et al. 2019; Murillo-A et al. 2022; Rivas-González et al. 2023; Zhang et al. 2024), the divergence times of the two superclades within Selaginellaceae, as revealed by this study and others (Arrigo et al. 2013; Klaus et al. 2017), occur more than 30 million years after the divergence of subg. *Selaginella* (Fig. 5, supplementary figs. S9 and S11, Supplementary Material online). The divergence of the *S. sanguinolenta* clade from each superclade also postdates their separation by over 20 million years (Fig. 5, supplementary figs. S9 and S11, Supplementary Material online), which does not support the scenario of rapid diversification. Therefore, we assume that ILS associated with rapid diversification, causing the conflicting topologies in the positions of the *S. sanguinolenta* clade, is less likely in this family.

The remarkably consistent split times between the *S. sanguinolenta* clade and superclades A or C, respectively, lend support to the allopolyploid origin hypothesis of the *S. sanguinolenta* clade (supplementary fig. S9, Supplementary Material online). Our molecular dating suggests that the allopolyploidization occurred in the Early Triassic, right after the Permian–Triassic extinction (Fig. 5a). While this was a period when over 90% of species on Earth went extinct, *Selaginella*, on the contrary, thrived (Pfefferkorn 2004; Visscher et al. 2004; Banks 2009). The allopolyploid origin was also supported by the result of the gene flow test (Fig. 3), the species network inference by the PhyloNet (Fig. 4a), the Ks2 peak in the interspecific Ks distribution (Fig. 3a, supplementary fig. S3, Supplementary Material online), and a clade-specific Ks peak in the Ks distribution of paralogous genes within the species of the *S. sanguinolenta* clade (supplementary fig. S2, Supplementary Material online). The plastome-based phylogenies showed that the *S. sanguinolenta* clade was sister to superclade C (Zhang et al. 2020; Chen et al. 2022; Zhang and Zhang 2022), which suggested that the common ancestor of superclade C was probably involved as a maternal side. The morphological and chromosomal evidence lent further support to the allopolyploid origin hypothesis of the *S. sanguinolenta* clade. The leaf morphology and leaf arrangement of the *S. sanguinolenta* clade were similar to those of superclade C, while the position of the rhizophore resembled that of superclade A (supplementary fig. S5, Supplementary Material online; supplementary table S6, Supplementary Material online). Their megaspore surfaces exhibiting an intermediate form between the two superclades and the triploid characterization of the *S. sanguinolenta* clade from the chromosome number provide compelling evidence for hybridization and polyploidization (supplementary figs. S6 and S8, Supplementary Material online). The intermediate spore sculptures and triploid chromosomes of the *S. sanguinolenta* clade were also reported previously (Takamiya 1993; Zhou et al. 2015b).

By integrating all the above evidence, we proposed that the enigmatic *S. sanguinolenta* clade originated by allopolyploidization during the Early Triassic following the Permian–Triassic mass extinction (Fig. 5a). This allopolyploidization event is postulated to coincide temporally with, or possibly precede, the epsilon (ε) polyploidy, a significant polyploidization event hypothesized to have arisen in the common ancestor of all angiosperms during the Triassic (Jiao et al. 2011; Wang et al. 2020b). In the context of land plants, polyploidization events known to precede this include the zeta (ζ) polyploidy, postulated to have occurred in the common ancestor of all seed plants in the Carboniferous period (Jiao et al. 2011), and another polyploidy event believed to have originated in the common ancestor of all leptosporangiates, excluding the Osmundales, during the Carboniferous–Permian transition (Huang et al. 2020). However, for all these events, it remains unresolved whether they resulted from autopolyploidization or allopolyploidization (Jiao et al. 2011; Huang et al. 2020; Wang et al. 2020b). Therefore, the inferred event leading to the origination of the *S*. *sanguinolenta* clade represents the earliest allopolyploidization in tracheophytes, to the best of our knowledge, and is supported by multiple lines of evidence.

Currently, evidence is not enough to illustrate the scenario of the ancient hybridization process. Based on the present distribution and habitats of subg. *Ruperstrae* (in superclade A), the *S. sinensis* clade (in superclade C), and the *S. sanguinolenta* clade, we tentatively speculated that the ancestral distributions of the three clades might have overlapped in the Induan stage (the current northern part of China and the Siberian region) during the Early Triassic (Fig. 5b), where the geological climate was predicted to be similar to that of the present time, with moderately low precipitation (Parrish et al. 1982; Barron and Fawcett 1995). The overlapping of ancestral distributions among these clades would have permitted their contact and hybridization. Ancient hybrid lineages have been defined as having stabilized genomes and subsequent diversification (Stull et al. 2023). The *S. sanguinolenta* clade demonstrates an excellent example of a paleo-allopolyploidization, which has recently diversified into several species (Fig. 5, supplementary figs. S9 and S11, Supplementary Material online). Remarkably, this clade has likely stabilized their genomes through the diploidization process, producing normal mega- and microspores (Takamiya 1993; Zhang et al. 2022), and the stabilization through the diploidization process is also evidenced by differentially fixed gene copies from the two parental lineages. A large proportion of the orthologous genes retain one of the two parental copies in the *S. sanguinolenta* clade. For example, the *S. sanguinolenta* genome keeps gene set B from superclade A and gene set C from superclade C. The two gene sets supported the sister relationship of the *S. sanguinolenta* clade with superclade A or with superclade C, respectively (Fig. 6). The other orthologous genes (130 genes in this study) are retained as polymorphic (gene copies from both putative parents are kept). During the long-term evolution, the lineage has diversified into multiple species, such as *S. sanguinolenta*, *S. rossii*, *S. nummularifolia*, *S. aitchisonii*, *S. jacquemontii*, *S. sajanensis*, and *S. baodongii*.

### Perspectives on Chromosomal Evolution, Reproductive Strategy, and Phylogenetic Positions

Our data from chromosome observations proved that species of the *S. sanguinolenta* clade have 30 chromosomes, different from other species of *Selaginella* (Takamiya 1993) (supplementary figs. S7 and S8, Supplementary Material online). Based on chromosome numbers reported in *Selaginella* species so far, the basic chromosome number in the genus is likely *x* = 9 or 10, with 8 only in the *Heterostachys* clade (=subg. *Heterostachys* sensu Zhou and Zhang) (Jermy et al. 1967; Zhukova and Petrovsky 1975; Love and Love 1975, 1976; Takamiya 1993; Mukhopadhyay and Goswami 1996). Approximately 10% of fern and lycophyte taxa occasionally produce unreduced spores for apomictic reproduction (Walker 1966, 1984), which may lead to polyploidization and significantly contribute to the diversification of tracheophytes (Soltis et al. 2009; Wood et al. 2009; Barker et al. 2016). Thirty chromosomes in the *S. sanguinolenta* clade may be a result of tetraploidization following hybridization between parents with *x* = 9 or 10 and subsequent loss of six or ten chromosomes by chromosome fusion. Alternatively, triploidization could have occurred through the fusion of 1n and 2n spores (micro- and megaspores) with *x* = 10. Karyotyping of *S. rossii*, a member of the *S*. *sanguinolenta* clade, conducted by Takamiya (1993), identified its karyotype as 4m + 2sm + 4(st + t), organized into three sets. With this observation, Takamiya (1993) suggested that *S. rossii* is triploid with a basic chromosome number of 10. Therefore, based on direct chromosome observations confirming the three-set chromosome composition (Takamiya 1993), the triploid origin of the *S. sanguinolenta* clade is more likely than the tetraploid origin.

The sterility of triploids is well known, e.g. bananas and watermelons (Griffiths et al. 2000). However, megaspores and microspores of the *S. sanguinolenta* clade seem to be normal (Liu et al. 1989; Xiao et al. 2002; Zhou and Zhang 2015; Yan et al. 2016; Vaganov et al. 2019). This raises the question of how the *S. sanguinolenta* clade reproduces successfully. Do they reproduce by spores or vegetatively? Despite their triploid origin generated by hybridization, the species in the *S. sanguinolenta* clade seem to have successfully evolved into a species complex that produces spores and has a wide distribution and high genetic diversity (Takamiya 1993; Zhang et al. 2013; Skaptsov et al. 2020). Species in the *S. sanguinolenta* clade are widely distributed on the Eurasian continent (Zhang et al. 2013). The extensive distribution of the *S. sanguinolenta* clade across Eurasia is more feasibly explained by the dispersal of spores capable of long distance, rather than limited vegetative reproduction through rhizophores. Vegetative reproduction seems unlikely to account for the extensive distribution observed (Pleasants and Wendel 1989; Silvertown 2008). Moreover, the *S. sanguinolenta* clade exhibits large genomic variations (Skaptsov et al. 2020). It has been reported that vegetative reproduction generally results in lower genetic diversity, like clones (Pleasants and Wendel 1989; Pfeiffer et al. 2006; Otálora et al. 2013). These features do not fit the expectations of a lineage that undergoes primarily vegetative reproduction. A future study with chromosome-level assembly addressing the chromosomal evolution of *Selaginella* species will be necessary to validate the triploid over the tetraploid origin of the *S. sanguinolenta* clade.

Despite the challenges in detecting ancient polyploidy and hybrid events, recent advances in data acquisition and analytical methodology of genomic data have facilitated the discovery of such events in *Juglans*, *Castanea*, *Hamamelis*, *Parthenocissus*, and *Vitis* (Zhang et al. 2019c; Ma et al. 2022; Yu et al. 2022; Zhou and Xiang 2022). A putative hybrid genome, inferred by phylogenetic incongruence, is likely formed by the differential loss or fixation of gene copies from the two parental progenitors, as found in this study of *Selaginella* and previous studies of other taxa (Zhang et al. 2019c; Ma et al. 2022; Yu et al. 2022). Species or lineages originated through hybridization initially possess gene copies from both paternal and maternal progenitors (Sang and Zhong 2000; Folk et al. 2018; Stull et al. 2023). Along with time, the loss of gene copies or alleles from one of the progenitors due to genetic drift or selection, coupled with the emergence and accumulation of mutations, complicates the detection of ancient hybridization events (Stull et al. 2023). Recently, three different phylogenetic positions (including two positions of sister relationships with each parental progenitor) of the species created by an ancient hybridization were reported in walnuts (Zhang et al. 2019c). The three positions observed in walnuts mirrored those observed in the *S. sanguinolenta* clade of the Selaginellaceae: one as the sister to the putatively paternal lineage, another as the sister to the putatively maternal lineage, and the final one as the sister to the clade including the paternal and maternal lineages (Zhang et al. 2019c) (Fig. 1). We speculate that the three positions observed in the *S. sanguinolenta* clade and walnuts may have been caused by different selective pressures on different gene sets (Fig. 6). For example, the genes supporting the sister relationship of the *S. sanguinolenta* clade and superclade A probably retained the allele from the common ancestor of superclade A (paternal lineage), and lost the allele from the other parent (maternal lineage) (case B in Fig. 6). However, the genes representing the topology, where the *S. sanguinolenta* clade is sister to both superclades, appear to have maintained alleles from each common ancestor of the two superclades. We proposed that this phenomenon may be related to gene conversion occurring between duplicated gene copies resulting from polyploidization. Gene conversion between gene copies duplicated through polyploidization has indeed been observed in various plants, such as *Oryza*, *Sorghum*, *Arachis*, *Gossypium*, *Brassica*, *Populus*, and *Salix* (Wang et al. 2009, 2011, 2022; Paterson et al. 2012; Zhuang et al. 2019; Shen et al. 2021; Wei et al. 2021). It is noted that partial gene-level conversion occurs more frequently than entire gene-level conversion (Wang et al. 2009, 2022; Shen et al. 2021; Wei et al. 2021), and gene copies undergone conversion exhibit different phylogenetic placements (Wang et al. 2009, 2022; Shen et al. 2021; Wei et al. 2021). Over time, these gene copies, modified through gene conversion, look like inheriting alleles in nearly equal proportions from the two parental lineages (case A in Fig. 6). Thus, we suggest that the third position, being in a sister relationship to the clade consisting of both parental progenitors, could be taken as a signature of ancient hybridization. This speculation is pending inspection through genetic comparisons in the future.

In conclusion, our comprehensive study strongly supported the hypothesis that the *S. sanguinolenta* clade originated from an ancient hybridization event followed by polyploidization in the early Triassic period. This is probably the earliest allopolyploidy event known to have been reported so far. Our study not only provides new insight into the evolutionary history of the Selaginellaceae, a key group in vascular plants, but also pushes the paleo-polyploidization record back to the early Mesozoic. Further efforts are required to elucidate the evolutionary dynamics underlying the phylogenetic incongruence within superclade C (subg. *Stachygynandrum*) and understand how the *S. sanguinolenta* clade succeeded in diploidization and diversification, following the allopolyploidization.

## Materials and Methods

### Taxon Sampling and Data Retrieval

A total of 46 accessions, representing 40 species (including two *Isoetes* as outgroups), were included in the study (supplementary table S8, Supplementary Material online). Thirty-three accessions of 28 *Selaginella* species were newly sampled and sequenced (supplementary table S8, Supplementary Material online). Among the remaining species, RNA-seq data for seven species were downloaded from the One Thousand Plant Transcriptomes Initiative (1KP; One Thousand Plant Transcriptomes Initiative 2019), and those of two species were downloaded from the Sequence Read Archive (SRA) database in NCBI GenBank (supplementary table S8, Supplementary Material online). In addition, whole-genome data of *S. moellendorffii* was used (Banks et al. 2011), and RNA-seq data of two *Isoetes* species from 1KP and NCBI GenBank were downloaded for the outgroup (supplementary table S8, Supplementary Material online). Based on the two recently proposed infrageneric classifications of the genus *Selaginella*, our sampling includes six out of the seven subgenera proposed by Weststrand and Korall (2016a), excluding subg. *Exaltatae*, and incorporates all six subgenera proposed by Zhou and Zhang (2015). However, we failed to include all sections proposed by Zhou and Zhang (2015), in which three subgenera, *Ericetorum*, *Heterostachys*, and *Stachygynandrum*, were divided into six, five, and seven sections, respectively. For the three subgenera, our sampling covered only four of six sections in subg. *Ericetorum* (excluding sections *Megalosporarum* and *Myosurus*), four of five sections in subg. *Heterostachys* (excluding sect. *Auriculate*), and five of seven sections in subg. *Stachygynandrum* (excluding sections *Austroamericanae* and *Heterophyllae*). To present the representativeness of the species used in this study, we have presented the number of species corresponding to the latest infrageneric classification, as well as the number of species and accessions included, in supplementary table S9, Supplementary Material online.

### RNA Sequencing and Assembly

For newly sequenced species, total RNA was extracted from mixed tissue samples of stems, leaves, and sporophylls of plants growing in the greenhouse of the Institute of Botany, Chinese Academy of Sciences. Paired-end reads of 2 × 150 were generated by the Illumina HiSeq4000 platform at Novogene Co. Ltd. (Beijing, China). The raw sequence reads, ranging from 5.8 to 16.0 GB, were trimmed using Trimmomatic (Bolger et al. 2014) (supplementary table S8, Supplementary Material online), and used for de novo assembly using Trinity v. 2.0.6 with default settings (Grabherr et al. 2011). Redundant nucleotide sequence removal was performed using CD-HIT v4.6 with a threshold of 0.98 (Fu et al. 2012). The remaining transcripts were converted into amino acid sequences using TransDecoder, and the longest peptide for each gene was selected as the potential unigenes.

### Orthologous Genes Selection

To select the orthologous genes, OrthoFinder v.1.1.8 was used for clustering the homologous gene groups (Emms and Kelly 2015). Among the clustered gene groups, paralogous gene removal was carefully performed in two independent steps. Redundant copies and putative paralogous genes were removed as described previously (Ran et al. 2018). In the first step, using 13 accessions covering the phylogenetic diversity in Selaginellaceae, if each species possesses at least one transcript and morphologically similar species form a clade (monophyletic), the homologous gene group was selected for the second step. All the data from 46 accessions were used for the second paralog removal. The two criteria in the first step were also applied in the second step, and the aligned length was considered. When the aligned length of the gene was shorter than 1,000 bp, the gene was excluded for further analyses. Although the species has only one copy of the gene, the possibility of a paralog arising from duplication and loss of the ortholog could not be completely ruled out (see supplementary fig. S12, Supplementary Material online for examples of selected and excluded genes). Therefore, we cautiously performed the phylogenetic reconstructions and checked whether morphologically similar species form a monophyletic group. For phylogenetic reconstruction of each putative orthologous gene group, sequence alignments were performed using MAFFT (Katoh and Standley 2013), and single gene trees were constructed using RAxML v. 8 (Stamatakis 2006) with 1,000 rapid bootstrap replicates and the automatic selection of the best-fit amino acid substitution model (PROTGAMMAAUTO). Data Analysis in Molecular Biology and Evolution (DAMBE) 7 was used to test the potential substitution saturation of the selected orthologous genes (Xia 2018). Detailed information on the 347 selected orthologous genes in this study is provided in supplementary table S2, Supplementary Material online.

### Phylogenetic Analyses

While selecting orthologous genes with phylogenetic reconstructions, three different positions of the *S. sanguinolenta* clade were discovered (A–C in supplementary fig. S12, Supplementary Material online). Two of the three positions were previously reported (supplementary fig. S12a and c, Supplementary Material online), and one position was newly found in this study (supplementary fig. S12b, Supplementary Material online). For further analyses, the selected orthologous genes were divided into three gene sets according to the position of the *S. sanguinolenta* clade. The amino acid sequences of each selected orthologous gene were aligned using MAFFT, and the alignments were manually inspected to exclude sequences of low quality, such as partial sequences. Phylogenetic analyses were performed using two approaches. For the supermatrix approach, in which all the aligned sequences of orthologous genes concatenate into one matrix, a Perl script, catfasta2phyml.pl (https://github.com/nylander/catfasta2phyml), was used to concatenate the aligned amino acid sequences of the selected orthologous genes. A total of three concatenated supermatrices for each gene set were prepared. Phylogenetic reconstructions of the three supermatrices were conducted using the ML method with RAxML, incorporating 1,000 rapid bootstrap replicates and employing the PROTGAMMAAUTO model. For the supertree approach, single gene trees were constructed using RAxML independently with 1,000 bootstrap replicates and the PROTGAMMAAUTO model, followed by coalescent-based analyses performed with ASTRAL (Mirarab et al. 2014). Single gene trees of the three divided gene sets were rooted using outgroup rooting with Newick Utilities v. 1.7.0 (Junier and Zdobnov 2010), specifically employing the “nw_reroot”, and coalescent-based analyses were performed for each divided gene set. The local posterior probabilities of the species tree were estimated using ASTRAL as node support. We used DensiTree (Bouckaert 2010) and Dendroscope v. 3.6.3 (Huson and Scornavacca 2012) to visualize and manipulate the results of phylogenetic analyses.

### Polyploidization Analyses

We analyzed distributions of synonymous substitution per synonymous site (Ks) to detect the allopolyploidization event within the *S. sanguinolenta* clade. We utilized three species from the *S. sanguinolenta* clade: *S. sanguinolenta*, *S. rossii*, and *S. nummularifolia*, comparing them with species representing each superclade: *S. vardei* for superclade A and *S. sinensis* for superclade C. For interspecific Ks distribution, we speculated that if the *S. sanguinolenta* clade resulted from hybridization between the common ancestors of the two superclades, we would observe an older peak indicating the genetic differentiation between the two superclades and a younger peak indicating the genetic differentiation between each superclade and its inherited genomic components in the hybrid origin clade. Conversely, if the *S. sanguinolenta* clade did not originate through hybridization, we would expect to see only one peak representing the genetic differentiation between each superclade and the *S. sanguinolenta* clade. We selected the longest transcript, which ranged from 201 to 26,999 bp, and translated it into its corresponding amino acid sequence from 45 transcriptome datasets and one genome dataset (*S. moellendorffii*) used in this study. Transcriptome data are known to contain a large number of redundant transcripts, such as alternatively spliced transcripts and false paralogs generated during assembly (Ren et al. 2018; Wang et al. 2019). These can affect the detection of WGDs through Ks distribution, similarly to recent gene duplications (Cui et al. 2006; Ren et al. 2018; Wang et al. 2019; Zwaenepoel et al. 2019). Therefore, removing these redundant sequences is a crucial and essential step in Ks distribution analysis using transcriptome data (Wang et al. 2019). To effectively remove these sequences, utilizing the blastp alignment method (Altschul et al. 1990), we filtered out sequences exhibiting high similarity (e-value < 1e^−5^ and identity > 90%) for each species to remove redundant gene pairs, which are not relevant to the paleo-polyploidization event in this study. Pairwise blastp alignments between each transcript pair were performed to construct a list of homologous gene pairs (e-value 1e-5, -max_target_seqs 1).

For the Ks distribution of paralogous genes within each species, we speculate that if the *S. sanguinolenta* clade was formed by allopolyploidization, species in this clade would exhibit a unique Ks peak that is not detected in the Ks distributions of species in the other two superclades. As with the interspecific Ks distribution analysis, the longest transcripts were extracted from each transcriptome, and then blastp was performed and filtered with the same conditions. We constructed a list of homologous gene pairs by performing the blastp alignment within each species of the five *Selaginella* species (e-value 1e-5, -max_target_seqs 1). We also analyzed the Ks distribution of the 347 orthologous genes used in the phylogenetic reconstruction to determine if they were associated with the allopolyploidization event. We construct a list of homologous gene pairs between species of the *S. sanguinolenta* clade and *S. vardei* or *S. sinensis*. For all analyses, the Ks value for each gene pair was estimated using a custom script available at https://github.com/JinfengChen/Scripts (Wang et al. 2010; Zhang et al. 2012). To account for the varied evolutionary rates across species in the genus *Selaginella*, we calibrated the Ks distribution of all chosen species in *Selaginella* and *Isoetes tegetiformans* using the cftool module in MATLAB. The minimal Ks observed between *S. wallacei* and *I. tegetiformans* (supplementary table S10, Supplementary Material online) served as a reference to calibrate the evolutionary rate of the included species within *Selaginella* (Wang et al. 2018b).

### Intergroup Gene Flow and Species Network Analyses

We used HyDe (Blischak et al. 2018) to assess the gene flow and hybrid origin of the *S. sanguinolenta* clade that showed incongruent phylogenetic placements. This tool is similar to the ABBA-BABA test (also known as the *D*-statistic) and has been shown to provide helpful information for detecting hybridization among three ingroups based on phylogenetic invariants (Blischak et al. 2018). To conduct the HyDe analysis, we used concatenated and aligned nucleotide sequences totaling 444,491 bp in length. Based on phylogenetic results, the 46 accessions included in this study were divided into an outgroup and three ingroups. The outgroup consisted of two *Isoetes* and *S. selaginoides*, with the latter included due to its sister relationship with other *Selaginella* species and its unique absence of rhizophore. The ingroups were defined as follows: superclade A, comprising the four subgenera and representing one of two putative parental groups; superclade C, which includes subg. *Stachygynandrum* as the second putative parental group; and the *S. sanguinolenta* clade, identified as the putative hybrid origin group. If hybridization is present among the three groups, the *P*-value is statistically significant (<0.05), and the inheritance probability (γ) is estimated to range between 0 and 1, indicating varying degrees of hybridization between the two putative parents. To visually demonstrate the statistical substantiation of detected hybridization, a violin plot was drawn using 1,000 bootstrap replicates with the violin package in R v.3.3.2 (R Development Core Team 2016), highlighting the variance and central tendency of the simulation results. Using the maximum pseudo-likelihood method, we inferred a species network that models incomplete lineage sorting and hybridization. The species network was inferred using PhyloNet v.3.8.0 (Than et al. 2008) with the command “InferNetwork_MPL”. Single gene trees of three different gene sets constructed using RAxML were equally used for this analysis (A–C in supplementary fig. S12, Supplementary Material online). The single gene trees were rooted using Newick Utilities. Network inference was conducted with settings of 0 to 3 reticulations, specifying the *S*. *sanguinolenta* clade as a hybrid species, and optimizing branch lengths and inheritance probabilities (γ) under the pseudo-likelihood. The inferred species network was displayed by Dendroscope v. 3.6.3.

### Morphology, Spore, and Chromosome Observations

Morphological observations were conducted through field observations and examination of specimens deposited in the China National Herbarium (PE). Additionally, specimen images from various herbaria, publicly available online via the Chinese Virtual Herbarium (https://www.cvh.ac.cn) and JSTOR Global Plants (https://plants.jstor.org), were utilized for observation. For species not observed in the field or through specimens, morphological characteristics, including spore morphology, were taken from the literature (Zhang et al. 2013; Zhou and Zhang 2015; Weststrand and Korall 2016a). The surface sculpture of spores is a key characteristic that exhibits distinct differences between different clades or species (Korall and Taylor 2006). To compare the surface sculpture of mega- and microspores between representative species of the putative hybrid origin clade (*S. sanguinolenta* clade) and the two putative parental clades (superclades A and C), we examined the surface sculpture of spores using scanning electron microscopy (SEM). Spores were collected from mature strobili of voucher specimens deposited in the herbarium PE or fresh individuals cultivated in the green house of the Institute of Botany, Chinese Academy of Sciences (IBCAS). Plant materials of *S. vardei* (representing superclade A) and *S. sanguinolenta* (representing the *S. sanguinolenta* clade) collected from Sichuan, and *S. sinensis* (representing superclade C) collected from Beijing were selected. Megaspores were affixed directly to a sample stub with double-sided adhesive tape, while unopened sporangia containing microspores were attached to the stub and smashed with a dissecting needle to release microspores. The sample stub was then sputter-coated with platinum. Observation and photography of a large number of spores were conducted for multiple individuals from each of the three species, using a Hitachi S-4800 (Tokyo, Japan) field emission SEM. Specifically, hundreds of mega- and microspores were observed, and over 20 photographs of megaspores and over ten photographs of microspores were taken, considering factors such as maturity, orientation, and angle.

To confirm whether 30 chromosomes are synapomorphies of the *S. sanguinolenta* clade and observe the chromosome numbers of the *S. sinensis* clade, fresh individuals of *S. sanguinolenta* and *S. sinensis* were used, which were grown in the green house of IBCAS. Vigorous root tips were fixed in a Carnoy I solution (3:1 ethanol to glacial acetic acid) at 4 °C for at least 3 h after being pretreated with an ice-water mixture in a dark room for 24 h. Dozens of fixed root tips were digested at 37 °C in a combination (1:1) of 2% cellulase and 2% pectinase for 60 min before staining with an improved carbol-fuchsin solution and being squashed for observation. The photographs of dozens of cells were taken using a Zeiss Axio Imager A1 camera. For other *Selaginella* species, beside *S. sanguinolenta* and *S. sinensis*, chromosome numbers and karyotypes were obtained from the literature (Jermy et al. 1967; Love and Love 1975, 1976; Zhukova and Petrovsky 1975; Takamiya 1993; Mukhopadhyay and Goswami 1996; VanBuren et al. 2018).

### Molecular Dating for the Ancient Hybridization

Currently available methods for molecular dating can only be applied to bifurcating species, but hybridization is not bifurcating. In this study, through phylogenetic analyses, species network inference, and gene flow test, we hypothesized that the *S. sanguinolenta* clade may have originated by hybridization between the common ancestors of two superclades A and C. Assuming that the *S. sanguinolenta* clade was indeed formed by hybridization between the common ancestors of these two superclades, we posited that the divergence times of the putative hybrid origin clade from each of the putative parental clades would be identical. Therefore, utilizing gene sets B and C, which show sister relationships with superclades A and C in phylogenetic analyses, respectively, we estimated the divergence times using gene sets B and C. If these divergence times were found to be identical, we inferred that time as the likely period during which hybridization occurred. The divergence time estimation for *Selaginella* species was performed using penalized likelihood (PL) implemented in r8s v.1.81 (Sanderson 2003) and MCMCtree implemented in PAML v.4.9 (Yang 2007). In both methods, the same two fossils were used as calibration points. The split between Isoëtaceae and Selaginellaceae was calibrated at 370 Ma based on the isoëtalean *Lepidosigillaria*, which is considered the earliest fossil of Isoëtales (rhizomorphic lycopsids), reported in the Late Devonian (Bateman et al. 1992; Kenrick and Crane 1997). Previously, Klaus et al. (2017) used isophyllous *Selaginella resimus* (Rowe 1988) to fix the split between the *S. sanguinolenta* clade and all other *Selaginella* species at 330 to 350 Ma based on the topology A in supplementary fig. S12, Supplementary Material online as the backbone, but the *S. sanguinolenta* clade is anisophyllous, which is morphologically inconsistent with the isophyllous fossil of *S. resimus*. Moreover, the *S. sanguinolenta* clade is hypothesized to have formed through hybridization between the common ancestors of superclades A and C; thus, the fossil of *S. resimus* is not appropriate to fix the split between the *S. sanguinolenta* clade and all other *Selaginella* species (including superclades A and C). Instead, the fossil of *S. suissei* found in the Late Carboniferous (ca. 304 Ma) (Zeiller 1906) was used to calibrate the split between subg. *Selaginella* (*S. selaginoides*) and the remaining *Selaginella* species. The fossil of *S. suissei* exhibited dimorphic leaves (anisophyllous) and a distinctive bisporangiate cone with apical microsporangia and basal megasporangia, similar to the living anisophyllous *Selaginella* species (Thomas 1992) and distinct from the subg. *Selaginella*.

Using the PL method, ML trees based on two concatenated matrices obtained by RAxML were used as input. Prior to estimating the divergence time, a cross-validation test was performed to determine the best smoothing value for each tree. With the best smoothing values, the divergence times were estimated with the TN and default settings of the other parameters. For the MCMCtree method, the rough substitution rates were estimated using the CODEML in PAML, and then the gradient (*g*) and Hessian (*H*) were estimated using the MCMCtree in PAML for each dataset. Finally, the divergence times were estimated using the calculated rgene_gamma and sigma2_gamma. The MCMC chain was analyzed for 10,000,000 generations with a sample frequency of 50 and a burn-in phase of 1,000,000 generations. We used Tracer v.1.7 (Rambaut et al. 2018) to check that the effective sample size was greater than 200. The estimated divergence times were displayed by FigTree v.1.4.3. Alternatively, we also conducted the divergence time estimation for Selaginellaceae using maternally inherited plastid genome data to compare whether the estimated divergence time between the *S. sanguinolenta* clade and superclade C is consistent with the result of gene set C. We used a concatenated dataset of 33 plastid gene sequences from 31 plastomes of *Selaginella* and *Isoetes*, which was used in a previous study (Zhang et al. 2020). The rough substitution rate was estimated using BASEML in PAML, after which the process was performed as above. We compared the estimated divergence times for splits between the *S. sanguinolenta* clade and the two superclades inferred from the two gene sets and plastome data independently, and then visualized the divergence time inferred from gene set C as the backbone of molecular dating for the hybridization occurrence.

## Supplementary Material

msae153_Supplementary_Data

## Data Availability

All relevant data can be found within the manuscript and its supporting information. Raw RNA sequencing data generated in this study are available at the Sequence Read Archive of NCBI GenBank, under BioProject PRJNA945812. The voucher specimens were deposited in the China National Herbarium (PE), and other data and materials provided in this manuscript are available from the corresponding author upon reasonable request.

## References

[msae153-B1] Altschul SF , Gish W, Miller W, Myers EW, Lipman DJ. Basic local alignment search tool. J Mol Biol. 1990:215(3):403–410. 10.1016/S0022-2836(05)80360-2. 2231712

[msae153-B2] Arrigo N , Therrien J, Anderson CL, Windham MD, Haufler CH, Barker MS. A total evidence approach to understanding phylogenetic relationships and ecological diversity in *Selaginella* subg. *Tetragonostachys*. Am J Bot. 2013:100(8):1672–1682. 10.3732/ajb.1200426. 23935110

[msae153-B3] Ash S . Late Triassic plants from the Chinle formation in north-eastern Arizona. Paleoontology. 1972:15:598–618.

[msae153-B4] Baniaga AE , Arrigo N, Barker MS. The small nuclear genomes of *Selaginella* are associated with a low rate of genome size evolution. Genome Biol Evol. 2016:8(5):1516–1525. 10.1093/gbe/evw091. 27189987 PMC4898805

[msae153-B5] Banks JA . *Selaginella* and 400 million years of separation. Annu Rev Plant Biol. 2009:60(1):223–238. 10.1146/annurev.arplant.59.032607.092851. 19575581

[msae153-B6] Banks JA , Nishiyama T, Hasebe M, Bowman JL, Gribskov M, dePamphilis C, Albert VA, Aono N, Aoyama T, Ambrose BA, et al The *Selaginella* genome identifies genetic changes associated with the evolution of vascular plants. Science. 2011:332(6032):960–963. 10.1126/science.1203810. 21551031 PMC3166216

[msae153-B7] Barker MS , Arrigo N, Baniaga AE, Li Z, Levin DA. On the relative abundance of autopolyploids and allopolyploids. New Phytol. 2016:210(2):391–398. 10.1111/nph.13698. 26439879

[msae153-B8] Barron EJ , Fawcett PJ. The climate of Pangaea: a review of climate model simulations of the permian. In: Scholle PA, Peryt TM, Ulmer-Scholle DS, editors. The permian of northern Pangea. Berlin, Heidelberg: Springer; 1995. p. 37–52.

[msae153-B9] Bateman RM , DiMichele WA, Willard DA. Experimental cladistic analysis of anatomically preserved arborescent Lycopsids from the Carboniferous of Euramerica: an essay on paleobotanical phylogenetics. Ann Missouri Bot Gard. 1992:79(3):500–559. 10.2307/2399752.

[msae153-B10] Blischak PD , Chifman J, Wolfe AD, Kubatko LS. HyDe: a python package for genome-scale hybridization detection. Syst Biol. 2018:67(5):821–829. 10.1093/sysbio/syy023. 29562307 PMC6454532

[msae153-B11] Bolger AM , Lohse M, Usadel B. Trimmomatic: a flexible trimmer for Illumina sequence data. Bioinformatics. 2014:30(15):2114–2120. 10.1093/bioinformatics/btu170. 24695404 PMC4103590

[msae153-B12] Bouckaert RR . DensiTree: making sense of sets of phylogenetic trees. Bioinformatics. 2010:26(10):1372–1373. 10.1093/bioinformatics/btq110. 20228129

[msae153-B13] Chen C , Ruhfel BR, Li J, Wang Z, Zhang L, Zhang L, Mao X, Wang J, He D, Luo Y, et al Phylotranscriptomics of Swertiinae (Gentianaceae) reveals that key floral traits are not phylogenetically correlated. J Integr Plant Biol. 2023:65(6):1490–1504. 10.1111/jipb.13464. 36749624

[msae153-B14] Chen S , Wang T, Shu J, Xiang QP, Yang T, Zhang XC, Yan Y. Plastid phylogenomics and plastome diversity of the extant lycophytes. Genes (Basel). 2022:13(7):1280. 10.3390/genes13071280. 35886063 PMC9316050

[msae153-B15] Cui L , Wall PK, Leebwens-Mack JH, Lindsay BG, Soltis DE, Doyle JJ, Soltis PS, Carlson JE, Arumuganathan K, Barakat A, et al Widespread genome duplications throughout the history of flowering plants. Genome Res. 2006:16(6):738–749. 10.1101/gr.4825606. 16702410 PMC1479859

[msae153-B16] Du XY , Lu JM, Li DZ. Extreme plastid RNA editing may confound phylogenetic reconstruction: a case study of *Selaginella* (lycophytes). Plant Diversity. 2020:42(5):356–361. 10.1016/j.pld.2020.06.009. 33134619 PMC7584784

[msae153-B17] Emms DM , Kelly S. OrthoFinder: solving fundamental biases in whole genome comparisons dramatically improves orthogroup inference accuracy. Genome Biol. 2015:16(1):157. 10.1186/s13059-015-0721-2. 26243257 PMC4531804

[msae153-B18] Feng S , Ru D, Sun Y, Mao K, Milne R, Liu J. Trans-lineage polymorphism and non-bifurcating diversification of the genus *Picea*. New Phytol. 2019:221(1):576–587. 10.1111/nph.15590. 30415488

[msae153-B19] Folk RA , Soltis PS, Soltis DE, Guralnick R. New prospects in the detection and comparative analysis of hybridization in the tree of life. Am J Bot. 2018:105(3):364–375. 10.1002/ajb2.1018. 29683488

[msae153-B20] Fu LM , Niu BF, Zhu ZW, Wu ST, Li WZ. CD-HIT: accelerated for clustering the next-generation sequencing data. Bioinformatics. 2012:28(23):3150–3152. 10.1093/bioinformatics/bts565. 23060610 PMC3516142

[msae153-B21] Goulet BE , Roda F, Hopkins R. Hybridization in plants: old ideas, new techniques. Plant Physiol. 2017:173(1):65–78. 10.1104/pp.16.01340. 27895205 PMC5210733

[msae153-B22] Grabherr MG , Haas BJ, Yassour M, Levin JZ, Thompson DA, Amit L, Adiconis X, Fan L, Raychowdhury R, Zeng Q, et al Full-length transcriptome assembly from RNA-Seq data without a reference genome. Nat Biotechnol. 2011:29(7):644–652. 10.1038/nbt.1883. 21572440 PMC3571712

[msae153-B23] Grant V . Plant speciation. New York: Columbia University Press; 1981.

[msae153-B24] Griffiths AJF , Miller JH, Suzuki DT, Lewontin RC, Gelbart WM. An introduction to genetic analysis. 7th ed. New York, USA: Freeman WH; 2000.

[msae153-B25] Hittinger CT , Johnston M, Tossberg JT, Rokas A. Leveraging skewed transcript abundance by RNA-Seq to increase the genomic depth of the tree of life. Proc Natl Acad Sci USA. 2010:107(4):1476–1481. 10.1073/pnas.0910449107. 20080632 PMC2824393

[msae153-B26] Huang CH , Qi X, Chen D, Qi J, Ma H. Recurrent genome duplication events likely contributed to both the ancient and recent rise of ferns. J Integr Plant Biol. 2020:62(4):433–455. 10.1111/jipb.12877. 31628713

[msae153-B27] Huang CH , Sun R, Hu Y, Zeng L, Zhang N, Cai L, Zhang Q, Koch MA, Al-Shehbaz I, Edger PP, et al Resolution of Brassicaceae phylogeny using nuclear genes uncovers nested radiations and supports convergent morphological evolution. Mol Biol Evol. 2016a:33(2):394–412. 10.1093/molbev/msv226. 26516094 PMC4866547

[msae153-B28] Huang CH , Zhang CF, Liu M, Hu Y, Gao TG, Qi J, Ma H. Multiple polyploidization events across Asteraceae with two nested events in the early history revealed by nuclear phylogenomics. Mol Biol Evol. 2016b:33(11):2820–2835. 10.1093/molbev/msw157. 27604225 PMC5062320

[msae153-B29] Huson DH , Scornavacca C. Dendroscope 3: an interactive tool for rooted phylogenetic trees and networks. Syst Biol. 2012:61(6):1061–1067. 10.1093/sysbio/sys062. 22780991

[msae153-B30] Jermy AC . Selaginellaceae. In: Kubitzki K, Kramer KU, Green PS, editors. The families and genera of vascular plants, pteridophytes and gymnosperms. Berlin: Springer; 1990. p. 39–45.

[msae153-B31] Jermy AC , Jones K, Colden C. Cytomorphological variation in *Selaginella*. J Linn Soc Bot. 1967:60(382):147–158. 10.1111/j.1095-8339.1967.tb00083.x.

[msae153-B32] Jiao Y , Wickett NJ, Ayyampalayam S, Chanderbali AS, Landherr L, Ralph PE, Tomsho LP, Hu Y, Liang H, Soltis PS, et al Ancestral polyploidy in seed plants and angiosperms. Nature. 2011:473(7345):97–100. 10.1038/nature09916. 21478875

[msae153-B33] Junier T , Zdobnov EM. The newick utilities: high-throughput phylogenetic tree processing in the UNIX shell. Bioinformatics. 2010:26(13):1669–1670. 10.1093/bioinformatics/btq243. 20472542 PMC2887050

[msae153-B34] Kang JS , Yu J, Zhang XC, Xiang QP. The associated evolution among the extensive RNA editing, GC-biased mutation, and PPR family expansion in the organelle genomes of Selaginellaceae. J Syst Evol. 2022:61(5):890–905. 10.1111/jse.12927.

[msae153-B35] Kang JS , Zhang HR, Wang YR, Liang SQ, Mao ZY, Zhang XC, Xiang QP. Distinctive evolutionary pattern of organelle genomes linked to the nuclear genome in Selaginellaceae. Plant J. 2020:104(6):1657–1672. 10.1111/tpj.15028. 33073395

[msae153-B36] Katoh K , Standley DM. MAFFT multiple sequence alignment software version 7: improvements in performance and usability. Mol Biol Evol. 2013:30(4):772–780. 10.1093/molbev/mst010. 23329690 PMC3603318

[msae153-B37] Kenrick P , Crane P. The origin and early diversification of land plants: a cladistic study. Washington (DC): Smithsonian Institution Press; 1997.

[msae153-B38] Klaus KV , Schulz C, Bauer DS, Stützel T. Historical biogeography of the ancient lycophyte genus *Selaginella*: early adaptation to xeric habitats on Pangea. Cladistics. 2017:33(5):469–480. 10.1111/cla.12184. 34724754

[msae153-B39] Korall P , Kenrick P. Phylogenetic relationships in Selaginellaceae based on *rbcL* sequences. Am J Bot. 2002:89(3):506–517. 10.3732/ajb.89.3.506. 21665649

[msae153-B40] Korall P , Kenrick P. The phylogenetic history of Selaginellaceae based on DNA sequences from the plastid and nucleus: extreme substitution rates and rate heterogeneity. Mol Phylogenet Evol. 2004:31(3):852–864. 10.1016/j.ympev.2003.10.014. 15120383

[msae153-B41] Korall P , Taylor WA. Megaspore morphology in the Selaginellaceae in a phylogenetic context: a study of the megaspore surface and wall structure using scanning electron microscopy. Grana. 2006:45(1):22–60. 10.1080/00173130500520453.

[msae153-B42] Kubatko LS , Chifman J. An invariants-based method for efficient identification of hybrid species from large-scale genomic data. BMC Evol Biol. 2019:19(1):112. 10.1186/s12862-019-1439-7. 31146685 PMC6543680

[msae153-B43] Li Z , Baniaga AE, Sessa EB, Scascitelli M, Graham SW, Rieseberg LH, Barker MS. Early genome duplications in conifers and other seed plants. Sci Adv. 2015:1(10):e1501084. 10.1126/sciadv.1501084. 26702445 PMC4681332

[msae153-B44] Liu BD , Bao WM, Aur CW. Studies on the spores of morphology of the family Selaginellaceae from China. Bull Bot Res. 1989:9:113–132.

[msae153-B45] Love A , Love D. In IOPB chromosome number reports XLVIII. Taxon. 1975:24:347–372.

[msae153-B46] Love A , Love D. In IOPB chromosome number reports LIII. Taxon. 1976:25(4):483–500. 10.1002/j.1996-8175.1976.tb00446.x.

[msae153-B47] Ma ZY , Nie ZL, Liu XQ, Tian JP, Zhou YF, Zimmer E, Wen J. Phylogenetic relationships, hybridization events, and drivers of diversification of East Asian wild grapes as revealed by phylogenomic analyses. J Syst Evol. 2022:61(2):273–283. 10.1111/jse.12918.

[msae153-B48] Maddison WP . Gene trees in species trees. Syst Biol. 1997:46(3):523–536. 10.1093/sysbio/46.3.523.

[msae153-B49] Mirarab S , Reaz R, Bayzid MS, Zimmermann T, Swenson MS, Warnow T. ASTRAL: genome-scale coalescent-based species tree estimation. Bioinformatics. 2014:30(17):i541–i548. 10.1093/bioinformatics/btu462. 25161245 PMC4147915

[msae153-B50] Moody ML , Rieseberg LH. Sorting through the chaff, nDNA gene trees for phylogenetic inference and hybrid identification of annual sunflowers (*Helianthus* sect. *Helianthus*). Mol Phylogenet Evol. 2012:64(1):145–155. 10.1016/j.ympev.2012.03.012. 22724134

[msae153-B51] Mower JP , Ma P, Grewe F, Taylor A, Michael TP, VanBuren R, Qiu YL. Lycophyte plastid genomics: extreme variation in GC, gene and intron content and multiple inversions between a direct and inverted orientation of the rRNA repeat. New Phytol. 2019:222(2):1061–1075. 10.1111/nph.15650. 30556907 PMC6590440

[msae153-B52] Mukhopadhyay R , Goswami N. Cytotaxonomic observations on two species of *Selaginella* P. Beauv. from India. Indian Fern J. 1996:13:22–29.

[msae153-B53] Murillo-A J , Valencia-D J, Orozco CI, Parra-O C, Neubig KM. Incomplete lineage sorting and reticulate evolution mask species relationships in Brunelliaceae, an Andean family with rapid, recent diversification. Am J Bot. 2022:109(7):1139–1156. 10.1002/ajb2.16025. 35709353

[msae153-B54] One Thousand Plant Transcriptomes Initiative . One thousand plant transcriptomes and the phylogenomics of green plants. Nature. 2019:574(7780):679–685. 10.1038/s41586-019-1693-2. 31645766 PMC6872490

[msae153-B55] Otálora MA , Salvador C, Martínez I, Aragón G. Does the reproductive strategy affect the transmission and genetic diversity of bionts in cyanolichens? A case study using two closely related species. Microb Ecol. 2013:65(2):517–530. 10.1007/s00248-012-0136-5. 23184157

[msae153-B56] Pamilo P , Nei M. Relationships between gene trees and species trees. Mol Biol Evol. 1988:5(5):568–583. 10.1093/oxfordjournals.molbev.a040517. 3193878

[msae153-B57] Parrish JT , Ziegler AM, Scotese CR. Rainfall patterns and the distribution of coals and evaporites in the Mesozoic and Cenozoic. Palaeogeogr Palaeoclimatol Palaeoecol. 1982:40(1-3):67–101. 10.1016/0031-0182(82)90085-2.

[msae153-B58] Paterson AH , Wendel JF, Gundlach H, Guo H, Jenkins J, Jin D, Llewellyn D, Showmaker KC, Shu S, Udall J, et al Repeated polyploidization of *Gossypium* genomes and the evolution of spinnable cotton fibres. Nature. 2012:492(7429):423–427. 10.1038/nature11798. 23257886

[msae153-B59] Pease JB , Haak DC, Hahn MW, Moyle LC. Phylogenomics reveals three sources of adaptive variation during a rapid radiation. PLoS Biol. 2016:14(2):e1002379. 10.1371/journal.pbio.1002379. 26871574 PMC4752443

[msae153-B60] Pfefferkorn HW . The complexity of mass extinction. Proc Natl Acad Sci USA. 2004:101(35):12779–12780. 10.1073/pnas.0404933101. 15328409 PMC516470

[msae153-B61] Pfeiffer T , Fritz S, Stech M, Frey W. Vegetative reproduction and clonal diversity in *Rhytidium rugosum* (Rhytidiaceae, Bryopsida) inferred by morpho-anatomical and molecular analyses. J Plant Res. 2006:119(2):125–135. 10.1007/s10265-005-0255-x. 16463068

[msae153-B62] Pleasants JM , Wendel JF. Genetic diversity in a clonal narrow endemic, *Erythronium propullans*, and in its widespread progenitor, *Erythronium albidum*. Amer J Bot. 1989:76(8):1136–1151. 10.1002/j.1537-2197.1989.tb15098.x.

[msae153-B63] Qi X , Kuo LY, Guo C, Li H, Li Z, Qi J, Wang L, Hu Y, Xiang J, Zhang C, et al A well-resolved fern nuclear phylogeny reveals the evolution history of numerous transcription factor families. Mol Phylogenet Evol. 2018:127:961–977. 10.1016/j.ympev.2018.06.043. 29981932

[msae153-B64] Rambaut A , Drummond AJ, Xie D, Baele G, Suchard MA. Posterior summarization in Bayesian phylogenetics using Tracer 1.7. Syst Biol. 2018:67(5):901–904. 10.1093/sysbio/syy032. 29718447 PMC6101584

[msae153-B65] Ran JH , Shen TT, Wang MM, Wang XQ. Phylogenomics resolves the deep phylogeny of seed plants and indicates partial convergent or homoplastic evolution between Gnetales and angiosperms. Proc R Soc B: Biol Sci. 2018:285(1881):20181012. 10.1098/rspb.2018.1012. PMC6030518 29925623

[msae153-B66] R Development Core Team . R: a language and environment for statistical computing, v.3.3.2. Vienna, Austria: R foundation for Statistical Computing; 2016. http://www.r-project.org.

[msae153-B67] Ren R , Wang H, Guo C, Zhang N, Zeng L, Chen Y, Ma H, Qi J. Widespread whole genome duplications contribute to genome complexity and species diversity in angiosperms. Mol Plant. 2018:11(3):414–428. 10.1016/j.molp.2018.01.002. 29317285

[msae153-B68] Rivas-González I , Rousselle M, Li F, Zhou L, Dutheil JY, Munch K, Shao Y, Wu D, Schierup MH, Zhang G. Pervasive incomplete lineage sorting illuminates speciation and selection in primates. Science. 2023:380(6648):eabn4409. 10.1126/science.abn4409. 37262154

[msae153-B69] Rowe NP . A herbaceous lycophyte from the lower carboniferous drybrook sandstone of the forest of Dean, Gloucestershire. Palaeontology. 1988:31:69–83.

[msae153-B70] Sanderson MJ . r8s: inferring absolute rates of molecular evolution and divergence times in the absence of a molecular clock. Bioinformatics. 2003:19(2):301–302. 10.1093/bioinformatics/19.2.301. 12538260

[msae153-B71] Sang T , Zhong Y. Testing hybridization hypotheses based on incongruent gene trees. Syst Biol. 2000:49(3):422–434. 10.1080/10635159950127321. 12116420

[msae153-B72] Shen H , Jin DM, Shu JP, Zhou XL, Lei M, Wei R, Shang H, Wei HJ, Zhang R, Liu L, et al Large-scale phylogenomic analysis resolves a backbone phylogeny in ferns. Gigascience. 2018:7(2):1–11. 10.1093/gigascience/gix116. PMC5795342 29186447

[msae153-B73] Shen S , Li Y, Wang J, Wei C, Wang Z, Ge W, Yuan M, Zhang L, Wang L, Sun S, et al Illegitimate recombination between duplicated genes generated from recursive polyploidizations accelerated the divergence of the genus *Arachis*. Genes (Basel). 2021:12(12):1944. 10.3390/genes12121944. 34946893 PMC8701993

[msae153-B74] Silvertown J . The evolutionary maintenance of sexual reproduction: evidence from the ecological distribution of asexual reproduction in clonal plants. Int J Plant Sci. 2008:169(1):157–168. 10.1086/523357.

[msae153-B75] Skaptsov MV , Vaganov AV, Kechaykin AA, Kutsev MG, Smirnov SV, Dorofeev VI, Borodina-Grabovskaya AE, Seregin AP, Sinitsina TA, Friesen NV, et al The cytotypes variability of the complex *Selaginella sanguinolenta* s. l. Turczaninowia. 2020:23(2):5–14. 10.14258/turczaninowia.23.2.1.

[msae153-B76] Soltis DE , Albert VA, Leebens-Mack J, Bell CD, Paterson AH, Zheng C, Sankoff D, DePamphilis CW, Wall PK, Soltis PS. Polyploidy and angiosperm diversification. Am J Bot. 2009:96(1):336–348. 10.3732/ajb.0800079. 21628192

[msae153-B77] Soltis PS , Soltis DE. The role of hybridization in plant speciation. Annu Rev Plant Biol. 2009:60(1):561–588. 10.1146/annurev.arplant.043008.092039. 19575590

[msae153-B78] Stamatakis A . RAxML-VI-HPC: maximum likelihood-based phylogenetic analyses with thousands of taxa and mixed models. Bioinformatics. 2006:22(21):2688–2690. 10.1093/bioinformatics/btl446. 16928733

[msae153-B79] Stebbins GL . Variation and evolution in plants. New York: Columbia University Press; 1950.

[msae153-B80] Stull GW , Pham KK, Soltis PS, Soltis DE. Deep reticulation: the long legacy of hybridization in vascular plant evolution. Plant J. 2023:114:743–766. 10.1111/tpj.16142. 36775995

[msae153-B81] Takamiya M . Comparative karyomorphology and interrelationships of *Selaginella* in Japan. J Plant Res. 1993:106(2):149–166. 10.1007/BF02344419.

[msae153-B82] Tang JY , Wei R, Zhang XC, Xiang QP. Mitogenome-based phylogenomics provides insights into the positions of the enigmatic *sinensis* group and the *sanguinolenta* group in Selaginellaceae (Lycophyte). Mol Phylogenet Evol. 2023:179:107673. 10.1016/j.ympev.2022.107673. 36528332

[msae153-B83] Than C , Ruths D, Nakhleh L. PhyloNet: a software package for analyzing and reconstructing reticulate evolutionary relationships. BMC Bioinformatics. 2008:9(1):322. 10.1186/1471-2105-9-322. 18662388 PMC2533029

[msae153-B84] Thomas BA . Paleozoic herbaceous Lycopsids and the beginnings of extant *Lycopodium* sens lat. and *Selaginella* sens. lat. Ann Missouri Bot Gard. 1992:79(3):623–631. 10.2307/2399756.

[msae153-B85] Vaganov AV , Shalimov AP, Kechaykin AA, Skaptsov MV, Smirnov SV, Sinitsyna TA, Kutsev MG, Zhang XC, Shmakov AI. Spore morphology of *Selaginella borealis*, *S. sanguinolenta* and *S. helvetica* (Selaginellaceae, Lycopodiophyta). Turczaninowia. 2019:22(2):142–150. 10.14258/turczaninowia.22.2.10.

[msae153-B86] VanBuren R , Wai CM, Ou S, Pardo J, Bryant D, Jiang N, Mockler TC, Edger P, Michael TP. Extreme haplotype variation in the desiccation-tolerant clubmoss *Selaginella lepidophylla*. Nat Commun. 2018:9(1):13. 10.1038/s41467-017-02546-5. 29296019 PMC5750206

[msae153-B87] Visscher H , Looy CV, Collinson ME, Brinkhuis H, Van Konijnenburg-Van Cittert JH, Kürschner WM, Sephton MA. Environmental mutagenesis during the end-Permian ecological crisis. Proc Natl Acad Sci USA. 2004:101(35):12952–12956. 10.1073/pnas.0404472101. 15282373 PMC516500

[msae153-B88] Walker TG . Apomixis and vegetative reproduction in ferns. In: Hawkes JG, editors. Reproductive biology and taxonomy of vascular plants. Oxford, UK: Pergamon Press Ltd; 1966. p. 152–161.

[msae153-B89] Walker TG . Chromosomes and evolution in pteridophytes. In: Sharma AK, Sharma A, editors. Chromosomes in evolution of eukaryotic groups, 2. Boca Raton (FL), USA: CRC Press; 1984. p. 103–141.

[msae153-B90] Wang J , Dong S, Yang LH, Harris AJ, Schneider H, Kang M. Allopolyploid speciation accompanied by gene flow in a tree fern. Mol Biol Evol. 2020a:37(9):2487–2502. 10.1093/molbev/msaa097. 32302390

[msae153-B91] Wang H , Guo C, Ma H, Qi J. Reply to Zwaenepoel et al.: meeting the challenges of detecting polyploidy events from transcriptome data. Mol Plant. 2019:12(2):137–140. 10.1016/j.molp.2018.12.020. 30599205

[msae153-B92] Wang J , Sun P, Li Y, Liu Y, Yang N, Yu J, Ma X, Sun S, Xia R, Liu X, et al An overlooked paleotetraploidization in Cucurbitaceae. Mol Biol Evol. 2018b:35(1):16–26. 10.1093/molbev/msx242. 29029269 PMC5850751

[msae153-B93] Wang X , Tang H, Bowers JE, Paterson AH. Comparative inference of illegitimate recombination between rice and sorghum duplicated genes produced by polyploidization. Genome Res. 2009:19(6):1026–1032. 10.1101/gr.087288.108. 19372385 PMC2694483

[msae153-B94] Wang X , Wang H, Wang J, Sun R, Wu J, Liu S, Bai Y, Mun JH, Bancroft I, Cheng F, et al The genome of the mesopolyploid crop species *Brassica rapa*. Nat Genet. 2011:43(10):1035–1039. 10.1038/ng.919. 21873998

[msae153-B95] Wang J , Yu J, Sun P, Li C, Song X, Lei T, Li Y, Yuan J, Sun S, Ding H, et al Paleo-polyploidization in Lycophytes. Genomics Proteomics Bioinformatics. 2020b:18(3):333–340. 10.1016/j.gpb.2020.10.002. 33157303 PMC7801247

[msae153-B96] Wang J , Zhang L, Wang J, Hao Y, Xiao Q, Teng J, Shen S, Zhang Y, Feng Y, Bao S, et al Conversion between duplicated genes generated by polyploidization contributes to the divergence of poplar and willow. BMC Plant Biol. 2022:22(1):298. 10.1186/s12870-022-03684-9. 35710333 PMC9205023

[msae153-B97] Wang D , Zhang Y, Zhang Z, Zhu J, Yu J. KaKs_Calculator 2.0: a toolkit incorporating gamma-series methods and sliding window strategies. Genomics Proteomics Bioinformatics. 2010:8(1):77–80. 10.1016/S1672-0229(10)60008-3. 20451164 PMC5054116

[msae153-B98] Wei C , Wang Z, Wang J, Teng J, Shen S, Xiao Q, Bao S, Feng Y, Zhang Y, Li Y, et al Conversion between 100-million-year-old duplicated genes contributes to rice subspecies divergence. BMC Genomics. 2021:22(1):460. 10.1186/s12864-021-07776-y. 34147070 PMC8214281

[msae153-B99] Wen J , Xiong Z, Nie ZL, Mao L, Zhu Y, Kan XZ, Ickert-Bond SM, Gerrath J, Zimmer EA, Fang XD. Transcriptome sequences resolve deep relationships of the grape family. PLoS One. 2013:8(9):e74394. 10.1371/journal.pone.0074394. 24069307 PMC3775763

[msae153-B100] Weststrand S , Korall P. A subgeneric classification of *Selaginella* (Selaginellaceae). Am J Bot. 2016a:103(12):2160–2169. 10.3732/ajb.1600288. 27999080

[msae153-B101] Weststrand S , Korall P. Phylogeny of Selaginellaceae: there is value in morphology after all!. Am J Bot. 2016b:103(12):1–24. 10.3732/ajb.1600156. 27999082

[msae153-B102] Wickett NJ , Mirarab S, Nguyen N, Warnow T, Carpenter E, Matasci N, Ayyampalayam S, Barker MS, Burleigh JG, Gitzendanner MA, et al Phylotranscriptomic analysis of the origin and early diversification of land plants. Proc Natl Acad Sci USA. 2014:111(45):E4859–E4868. 10.1073/pnas.1323926111. 25355905 PMC4234587

[msae153-B103] Wood TE , Takebayashi N, Barker MS, Mayrose I, Greenspoon PB, Rieseberg LH. The frequency of polyploid speciation in vascular plants. Proc Natl Acad Sci USA. 2009:106(33):13875–13879. 10.1073/pnas.0811575106. 19667210 PMC2728988

[msae153-B104] Xia X . DAMBE7: new and improved tools for data analysis in molecular biology and evolution. Mol Biol Evol. 2018:35(6):1550–1552. 10.1093/molbev/msy073. 29669107 PMC5967572

[msae153-B105] Xiang Y , Huang CH, Hu Y, Wen J, Li S, Yi T, Chen H, Xiang J, Ma H. Evolution of Rosaceae fruit types based on nuclear phylogeny in the context of geological times and genome duplication. Mol Biol Evol. 2017:34(2):262–281. 10.1093/molbev/msw242. 27856652 PMC5400374

[msae153-B106] Xiang QP , Tang JY, Yu JG, Smith DR, Zhu YM, Wang YR, Kang JS, Yang J, Zhang XC. The evolution of extremely diverged plastomes in Selaginellaceae (lycophyte) is driven by repeat patterns and the underlying DNA maintenance machinery. Plant J. 2022:111(3):768–784. 10.1111/tpj.15851. 35648423

[msae153-B107] Xiao XY , Lin RC, Chang CY, Chen XD. The comparative studies on microspores of fourteen species in *Selaginella*. Chin J Pharm Anal. 2002:22:20–24.

[msae153-B108] Xu Z , Xin T, Bartels D, Li Y, Gu W, Yao H, Liu S, Yu H, Pu X, Zhou J, et al Genome analysis of the ancient tracheophyte *Selaginella tamariscina* reveals evolutionary features relevant to the acquisition of desiccation tolerance. Mol Plant. 2018:11(7):983–994. 10.1016/j.molp.2018.05.003. 29777775

[msae153-B109] Yan D , Wang LJ, Song YY, Wang L, Du TT, Liu JX. Microspore morphology of Selaginellaceae in China and its systematic significance. Plant Syst Evol. 2016:302(5):561–574. 10.1007/s00606-016-1284-8.

[msae153-B110] Yang Z . PAML 4: phylogenetic analysis by maximum likelihood. Mol Biol Evol. 2007:24(8):1586–1591. 10.1093/molbev/msm088. 17483113

[msae153-B111] Yu J , Niu Y, You Y, Cox CJ, Barrett RL, Trias-Blasi A, Guo J, Wen J, Liu L, Chen Z. Integrated phylogenomic analyses unveil reticulate evolution in *Parthenocissus* (Vitaceae), highlighting speciation dynamics in the Himalayan–Hengduan Mountains. New Phytol. 2022:238(2):888–903. 10.1111/nph.18580. 36305244

[msae153-B112] Zeiller R . Bassin houiller et permien de Blanzy et du Creusot. Fasc. 2. Flore fossile. Texte: Imprimerie Nationale; 1906.

[msae153-B113] Zeng L , Zhang Q, Sun R, Kong H, Zhang N, Ma H. Resolution of deep angiosperm phylogeny using conserved nuclear genes and estimates of early divergence times. Nat Commun. 2014:5(1):4956. 10.1038/ncomms5956. 25249442 PMC4200517

[msae153-B114] Zhang Z , Liu G, Li M. Phylotranscriptomic discordance is best explained by incomplete lineage sorting within *Allium* subgenus *Cyathophora* and thus hemiplasy accounts for interspecific trait transition. Plant Diversity. 2024:46(1):28–38. 10.1016/j.pld.2023.07.004. 38343588 PMC10851291

[msae153-B115] Zhang XC , Nooteboom HP, Kato M. Selaginellaceae. In: Wu ZY, Raven PH, Hong DY, editors. Flora of China. St. Louis (Missouri), USA: Missouri Botanical Garden Press; 2013. p. 37–66.

[msae153-B116] Zhang HR , Wei R, Xiang QP, Zhang XC. Plastome-based phylogenomics resolves the placement of the *sanguinolenta* group in the spikemoss of lycophyte (Selaginellaceae). Mol Phylogenet Evol. 2020:147:106788. 10.1016/j.ympev.2020.106788. 32173413

[msae153-B117] Zhang HR , Xiang QP, Zhang XC. The unique evolutionary trajectory and dynamic conformations of DR and IR/DR-coexisting plastomes of the early vascular plant Selaginellaceae (Lycophyte). Genome Biol Evol. 2019a:11(4):1258–1274. 10.1093/gbe/evz073. 30937434 PMC6486807

[msae153-B118] Zhang Z , Xiao J, Wu J, Zhang H, Liu G, Wang X, Dai L. ParaAT: a parallel tool for constructing multiple protein-coding DNA alignments. Biochem Biophys Res Commun. 2012:419(4):779–781. 10.1016/j.bbrc.2012.02.101. 22390928

[msae153-B119] Zhang BW , Xu LL, Li N, Yan PC, Jiang XH, Woeste KE, Lin K, Renner SS, Zhang DY, Bai WN. Phylogenomics reveals an ancient hybrid origin of the Persian walnut. Mol Biol Evol. 2019c:36(11):2451–2461. 10.1093/molbev/msz112. 31163451

[msae153-B120] Zhang MH , Zhang XC. Integrative species delimitation of *Selaginella labordei* and closely related species: uncovering the mysterious identity of *S. jugorum* and *S. tibetica*, and description of a new species. Taxon. 2022:71(6):1155–1169. 10.1002/tax.12800.

[msae153-B121] Zhang MH , Zhang XC, Shalimov AP, Shmakov AI, Xiang QP. Integrative species delimitation of the *Selaginella sanguinolenta* (Selaginellaceae) group with description of a new species, *S. baodongii*. Taxon. 2023:72(6):1228–1243. 10.1002/tax.13082.

[msae153-B122] Zhang HR , Zhang XC, Xiang QP. Directed repeats co-occur with few short-dispersed repeats in plastid genome of a spikemoss, *Selaginella vardei* (Selaginellaceae, Lycopodiopsida). BMC Genomics. 2019b:20(1):484. 10.1186/s12864-019-5843-6. 31185895 PMC6560725

[msae153-B123] Zhang L , Zhu X, Zhao Y, Guo J, Zhang T, Huang W, Huang J, Hu Y, Huang CH, Ma H. Phylotranscriptomics resolves the phylogeny of Pooideae and uncovers factors for their adaptive evolution. Mol Biol Evol. 2022:39(2):msac026. 10.1093/molbev/msac026. 35134207 PMC8844509

[msae153-B124] Zhou XM , Jiang LJ, Zhang L, Gao XF, He ZR, Zhang LB. Spore morphology of *Selaginella* (Selaginellaceae) from China and its systematic significance. Phytotaxa. 2015b:237(1):001–067. 10.11646/phytotaxa.237.1.1.

[msae153-B125] Zhou XM , Rothfels CJ, Zhang L, He ZR, Péchon TL, He H, Lu NT, Knapp R, Lorence D, He XJ, et al A large-scale phylogeny of the lycophyte genus *Selaginella* (Selaginellaceae: Lycopodiopsida) based on plastid and nuclear loci. Cladistics. 2015a:32(4):360–389. 10.1111/cla.12136. 34740298

[msae153-B126] Zhou W , Xiang QY. Phylogenomics and biogeography of *Castanea* (chestnut) and *Hamamelis* (witch-hazel)—choosing between RAD-seq and Hyb-Seq approaches. Mol Phylogenet Evol. 2022:176:107592. 10.1016/j.ympev.2022.107592. 35926825

[msae153-B127] Zhou XM , Zhang LB. A classification of *Selaginella* (Selaginellaceae) based on molecular (chloroplast and nuclear), macromorphological, and spore features. Taxon. 2015:64(6):1117–1140. 10.12705/646.2.

[msae153-B128] Zhou XM , Zhao J, Yang JJ, Le Péchon T, Zhang L, He ZR, Zhang LB. Plastome structure, evolution, and phylogeny of *Selaginella*. Mol Phylogenet Evol. 2022:169:107410. 10.1016/j.ympev.2022.107410. 35031459

[msae153-B129] Zhuang W , Chen H, Yang M, Wang J, Pandey MK, Zhang C, Chang WC, Zhang L, Zhang X, Tang R, et al The genome of cultivated peanut provides insight into legume karyotypes, polyploid evolution and crop domestication. Nat Genet. 2019:51(5):865–876. 10.1038/s41588-019-0402-2. 31043757 PMC7188672

[msae153-B130] Zhukova PG , Petrovsky VV. Chromosome numbers of some Western Chukolka plant species. Bot Zhurn (Moscow & Leningrad). 1975:60:395–401.

[msae153-B131] Zwaenepoel A , Li Z, Lohaus R, Van de Peer Y. Finding evidence for whole genome duplications: a reappraisal. Mol Plant. 2019:12(2):133–136. 10.1016/j.molp.2018.12.019. 30599206

