# Supplementary material for: The Possible Earliest Allopolyploidization in Tracheophytes Revealed by Phylotranscriptomics and Morphology of Selaginellaceae
Source: Mol Biol Evol. 2024 Aug 5;41(8):msae153. doi: 10.1093/molbev/msae153 (PMC11299036; doi:10.1093/molbev/msae153)
Supplement: msae153_Supplementary_Data [file msae153_supplementary_data.zip › Supporting Information_Figures_R2_20240715.pdf]

Supporting Information of

The possible earliest allopolyploidization in tracheophytes revealed by phylotranscriptomics and morphology of Selaginellaceae

Jong-Soo Kang, Ji-Gao Yu, Qiao-Ping Xiang, Xian-Chun Zhang

Figure S1. Maximum likelihood trees based on concatenated data sets of each gene set. (A) ML tree based on 130 genes of gene set A. (B) ML tree based on 81 genes of gene set B. (C) ML tree based on 136 genes of gene set C. Branch support values from RAxML are 100% unless otherwise indicated in red. Clades marked in different colors represent each subgenus or clade. Grey: genus *Isoetes*, Dark yellow: subg. *Selaginella*, Black: *S. sanguinolenta* clade, Pink: subg. *Repestrae*, Orange: subg. *Lepidophyllae*, Blue: subg. *Ericetorum*, Green: subg. *Gymnogynum*, Light purple: *S. sinensis* clade, Red: *Stachygynandrum* clade (= subg. *Stachygynandrum sensu* Zhou and Zhang), Light yellow: *S. pulvinata* clade (subg. *Pulviniella sensu* Zhou and Zhang), Purple: *Heterostachys* clade (= subg. *Heterostachys sensu* Zhou and Zhang).

Figure S2. Ks distribution of paralogous genes within each species. *Selaginella sanguinolenta*, *S. nummularifolia*, and *S. rossii* belong to the putative allopolyploid origin clade (*S. sanguinolenta* clade). *Selaginella vardei* and *S. sinensis* represent superclades A and C, respectively. The Ks gene pairs came from the best-match gene pair of the blast result and were calculated. (A) Gaussian fitting of the Ks distribution using the cftool module in Matlab. The distribution shows the Ks values fitted with a Gaussian model, highlighting the overall pattern of gene duplications. (B) Histograms of the raw Ks distribution without any cut-off applied. The Ks distribution was drawn using all gene pairs without any cut-offs. (C) Histograms of the Ks distribution after applying a 90% similarity cut-off. Gene pairs with more than 90% similarity were removed to exclude the putative recently duplicated gene copies, further eliminating the impact on detecting ancient whole-genome duplication patterns relevant to paleo-allopolyploidization in this study.

Figure S3. Interspecific Ks distribution between the *S. sanguinolenta* clade and each parental clade. *Selaginella sanguinolenta*, *S. nummularifolia*, and *S. rossii* belong to the putative allopolyploid origin clade (*S. sanguinolenta* clade). *Selaginella vardei* and *S. sinensis* represent superclades A and C, respectively. The Ks gene pairs came from the best-match gene pair of the blast result and were calculated. (A) Histograms of the raw Ks distribution without any cut-off applied. The Ks distribution was drawn using all gene pairs from interspecific comparisons without any cut-off. (B) Histograms of the Ks distribution after applying a 90% similarity cut-off for each species. Gene pairs with more than 90% similarity were removed to exclude the putative recently duplicated gene copies, further eliminating the impact on detecting ancient whole-genome duplication patterns relevant to paleo-allopolyploidization in this study. The Gaussian fitting of the Ks distribution is shown in Figure 3A.

Figure S4. Ks distribution of 347 orthologous genes used for phylogenetic reconstruction. Gaussian fitting of Ks distribution using cftool module in Matlab.

Figure S5. Rhizophores on dorsal side in the *Selaginella sanguinolenta* clade. (A and B) Rhizophores of *Selaginella rossii*. (C) Rhizophore of *Selaginella sanguinolenta*. The white dashed circle presents the

rhizophore on the dorsal side.

Figure S6. Megaspores and microspores of three representative *Selaginella* species. *S. vardei* belongs to subg. *Rupestrae* (superclade A), *S. sanguinolenta* represents the *S. sanguinolenta* clade, and *S. sinensis* belongs to subg. *Stachygynandrum* (superclade C).

Figure S7. Chromosome evolution of the Selaginellaceae. A species network inferred from nuclear single-gene trees was used as the backbone. The putative ancestral chromosome numbers of each clade were presented on the branches. The observed chromosome numbers and karyotypes of each species from this study and previous studies were presented on the right side of the species network. a: This study, b: Takamiya (1993), c: Jermy et al. (1967), d: VanBuren et al. (2018), e: Mukhopadhyay and Goswami (1996), f: Zhukova and Petrovsky (1975), g: Love and Love (1975), h: Love and Love (1976).

Figure S8. Somatic chromosomes of *Selaginella sanguinolenta* and *S. sinensis*. A-D: Somatic chromosomes at metaphase in *S. sanguinolenta*. E and F: Somatic chromosomes at metaphase in *S. sinensis*

Figure S9. Divergence time estimations for the Selaginellaceae using two different methods. The red stars indicate fossil calibration points. Divergence times were estimated using gene set B (left) and gene set C (right). Red arrows indicate the split between the *S. sanguinolenta* group and superclade A or C, respectively. (A) Divergence time estimated by Penalized likelihood (PL) method. (B) Divergence time estimated by MCMCtree method.

Figure S10. Conceptual differences between hybridization and incomplete lineage sorting. Both concepts of hybridization and incomplete lineage sorting were adopted from Sang and Zhong (2000) and Pamilo and Nei (1988). Representative species trees, where "a", "b", and "c" are ingroup species and o is an outgroup species. t_0 indicate time of speciation between ingroup and outgroup. t_f, t_g, t_i, t_j, t_k, and t_m indicate divergence times, and t_{h1} and t_{h2} indicate time when the lineages that hybridized to give rise to "b" diverged from "a" and "c". Representative species trees are given in (a, b). The two different gene trees are given in (c, d, e, and f). Expected gene-wise frequencies in the genome shown as near trees (b–f).

Figure S11. Divergence time estimation for the Selaginellaceae using plastid genome data. The red stars indicate fossil calibration points. Divergence time was estimated by MCMCtree method based on plastid genome data (Zhang et al., 2020). Red arrow indicates the split between the *S. sanguinolenta* group and superclade C.

Figure S12. Examples of selected and excluded single gene trees in this study. *Out*: outgroup, *Se*: subg. *Selaginella*, *Er*: subg. *Ericetorum*, *Gy*: subg. *Gymnogynum*, *Le*: subg. *Lepidophyllae*, *Ru*: subg. *Rupestrae*, *St*: *Stachygynandrum* clade, *He*: *Heterostachys* clade, *Pu*: *S. pulvinata* clade, *Sa*: *S. sanguinolenta* clade, *Si*: *S. sinensis* clade. *He*, *Pu*, *Sa*, and *Si* belong to subg. *Stachygynandrum* in the latest classification (Weststrand and Korall, 2016a). Single gene tree showing monophyletic for each lineage was selected for further analyses (A-C). Single gene tree showing nonmonophyletic in some lineage was excluded (D-F).

Table S1. Substitution saturation test for 347 orthologous genes using DAMBE. Iss: Index of substitution saturation; Iss.c: Index of substitution saturation assuming a symetrical true tree; Prob: Probability of significant difference between Iss and Iss.c. (two-tailed test).

Table S2. Functional information of the nuclear genes used for reconstructing phylogenies in this study.

Table S3. Fitting the Ks distribution of paralogous genes within each species by Gaussian model. *S.*

sanguinolenta, *S. nummularifolia*, and *S. rossii* belong to the *S. sanguinolenta* clade. *S. vardei* and *S. sinensis* represent superclades A and C, respectively.

Table S4. Fitting the interspecific Ks distribution among two superclades and *S. sanguinolenta* clade by Gaussian model. *S. sanguinolenta*, *S. nummularifolia*, and *S. rossii* belong to the *S. sanguinolenta* clade. *S. vardei* and *S. sinensis* represent superclades A and C, respectively.

Table S5. Fitting the Ks distribution of 347 orthologous genes used for phylogenetic reconstruction by Gaussian model. *S. sanguinolenta*, *S. nummularifolia*, and *S. rossii* belong to the *S. sanguinolenta* clade. *S. vardei* and *S. sinensis* represent superclades A and C, respectively.

Table S6. Morphological and chromosomal characters for each group within Selaginellaceae.

Table S7. Ninety-five percent highest posterior density (HPD) estimates for the age of split between superclades and occurrence of the allopolyploidization event within Selaginellaceae.

Table S8. List of 46 accessions and their information used in this study.

Table S9. Three representative infrageneric classifications in the genus *Selaginella* and representativeness of our sampling across the genus.

Table S10. The Ks values between *Selaginella* and *Isoetes*.

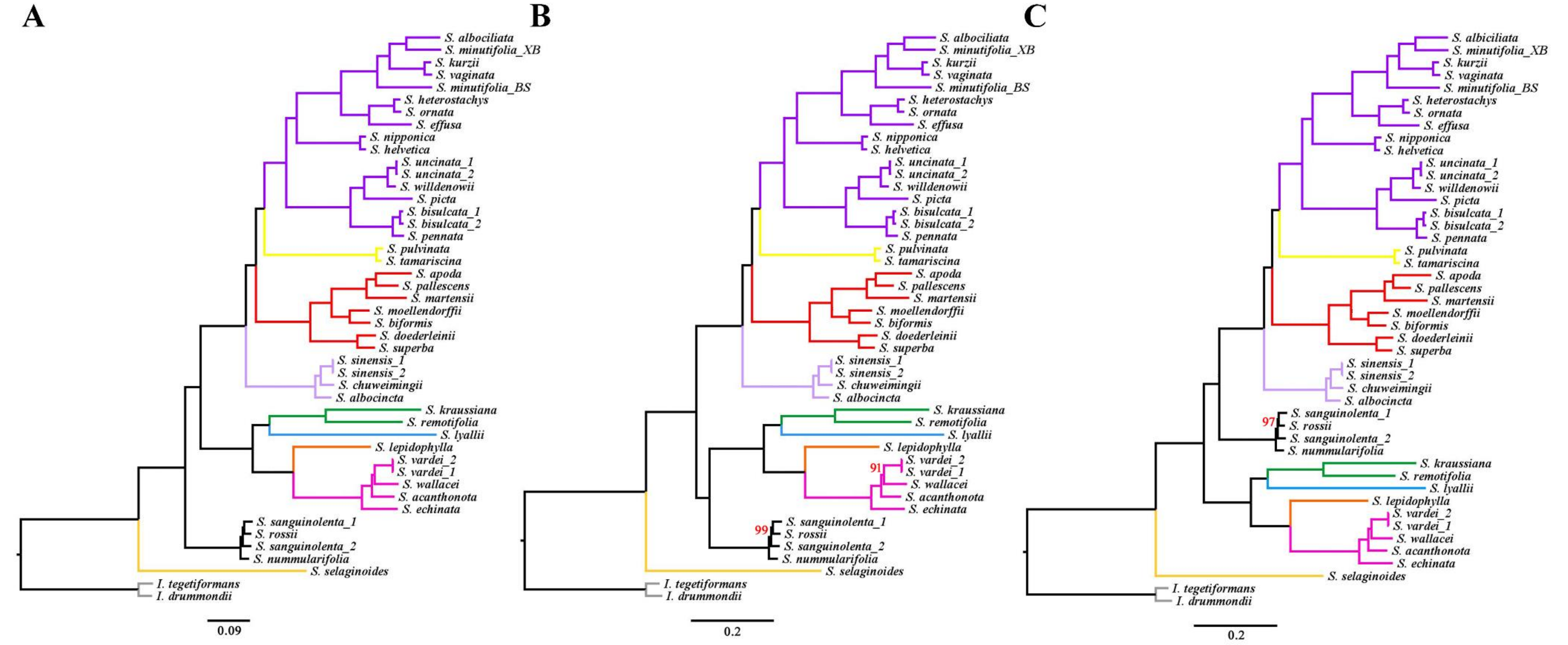

Figure S1. Maximum likelihood trees based on concatenated data sets of each gene set. (A) ML tree based on 130 genes of gene set A. (B) ML tree based on 81 genes of gene set B. (C) ML tree based on 136 genes of gene set C. Branch support values from RAxML are 100% unless otherwise indicated in red. Clades marked in different colors represent each subgenus or clade. Grey: genus *Isoetes*, Dark yellow: subg. *Selaginella*, Black: *S. sanguinolenta* clade, Pink: subg. *Repestrae*, Orange: subg. *Lepidophyllae*, Blue: subg. *Ericetorum*, Green: subg. *Gymnogynum*, Light purple: *S. sinensis* clade, Red: *Stachygynandrum* clade (= subg. *Stachygynandrum sensu* Zhou and Zhang), Light yellow: *S. pulvinata* clade (subg. *Pulviniella sensu* Zhou and Zhang), Purple: *Heterostachys* clade (= subg. *Heterostachys sensu* Zhou and Zhang).

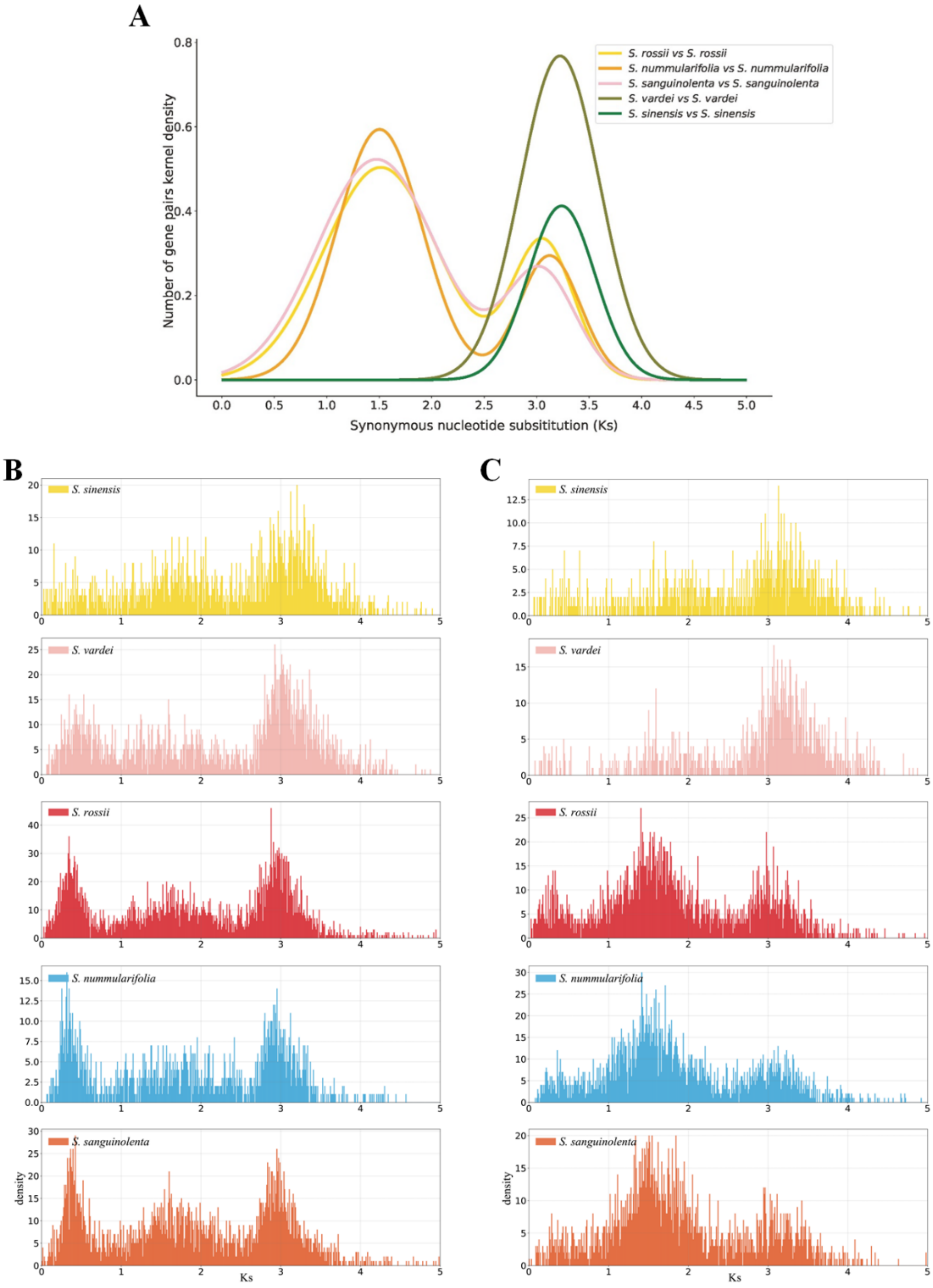

Figure S2. Ks distribution of paralogous genes within each species. *Selaginella sanguinolenta*, *S. nummularifolia*, and *S. rossii* belong to the putative allopolyploid origin clade (*S. sanguinolenta* clade). *Selaginella vardei* and *S. sinensis* represent superclades A and C, respectively. The Ks gene pairs came from the best-match gene pair of the blast result and were calculated. (A) Gaussian fitting of the Ks distribution using the cftool module in Matlab. The distribution shows the Ks values fitted with a Gaussian model, highlighting the overall pattern of gene duplications. (B) Histograms of the raw Ks distribution without any cut-off applied. The Ks distribution was drawn using all gene pairs without any cut-offs. (C) Histograms of the Ks distribution after applying a 90% similarity cut-off. Gene pairs with more than 90% similarity were removed to exclude the putative recently duplicated gene copies, further eliminating the impact on detecting ancient whole-genome duplication patterns relevant to paleo-allopolyploidization in this study.

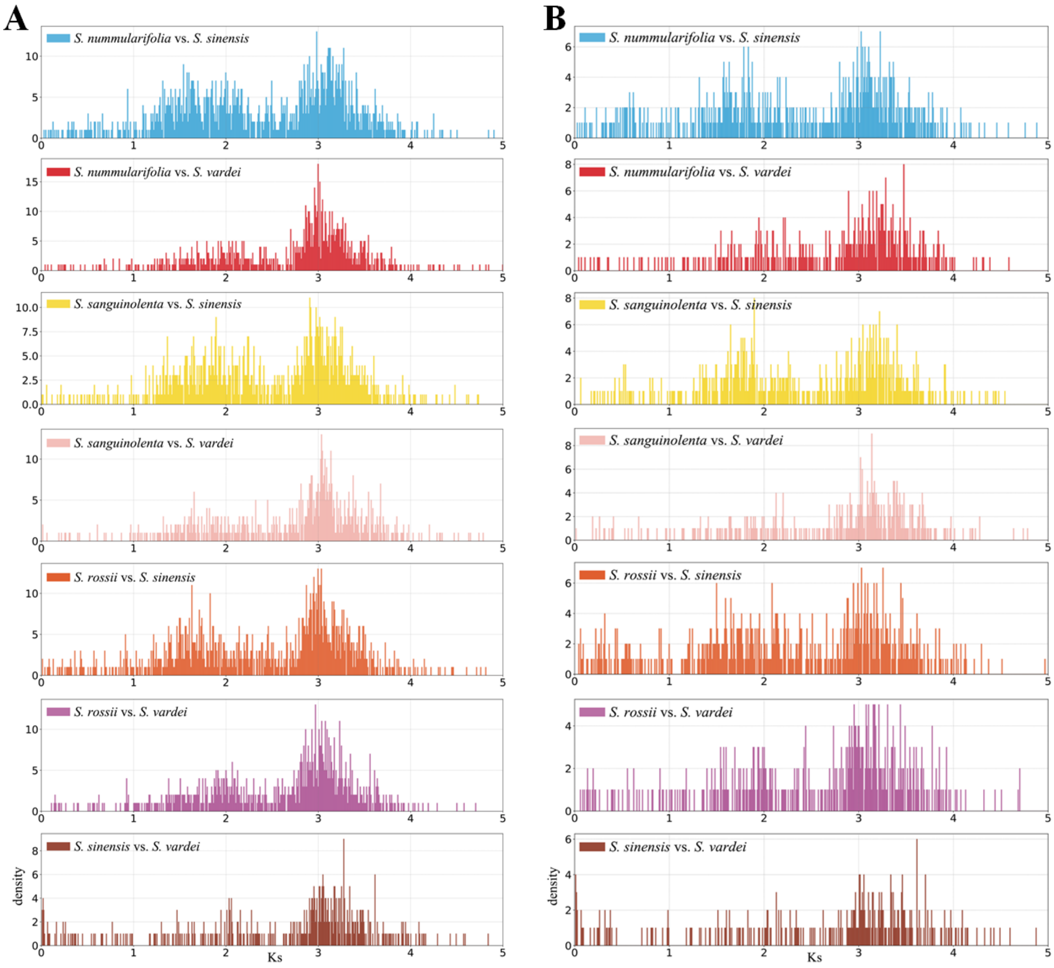

Figure S3. Interspecific Ks distribution between the *S. sanguinolenta* clade and each parental clade. *Selaginella sanguinolenta*, *S. nummularifolia*, and *S. rossii* belong to the putative allopolyploid origin clade (*S. sanguinolenta* clade). *Selaginella vardei* and *S. sinensis* represent superclades A and C, respectively. The Ks gene pairs came from the best-match gene pair of the blast result and were calculated. (A) Histograms of the raw Ks distribution without any cut-off applied. The Ks distribution was drawn using all gene pairs from interspecific comparisons without any cut-off. (B) Histograms of the Ks distribution after applying a 90% similarity cut-off for each species. Gene pairs with more than 90% similarity were removed to exclude the putative recently duplicated gene copies, further eliminating the impact on detecting ancient whole-genome duplication patterns relevant to paleo-allopolyploidization in this study. The Gaussian fitting of the Ks distribution is shown in Figure 3A.

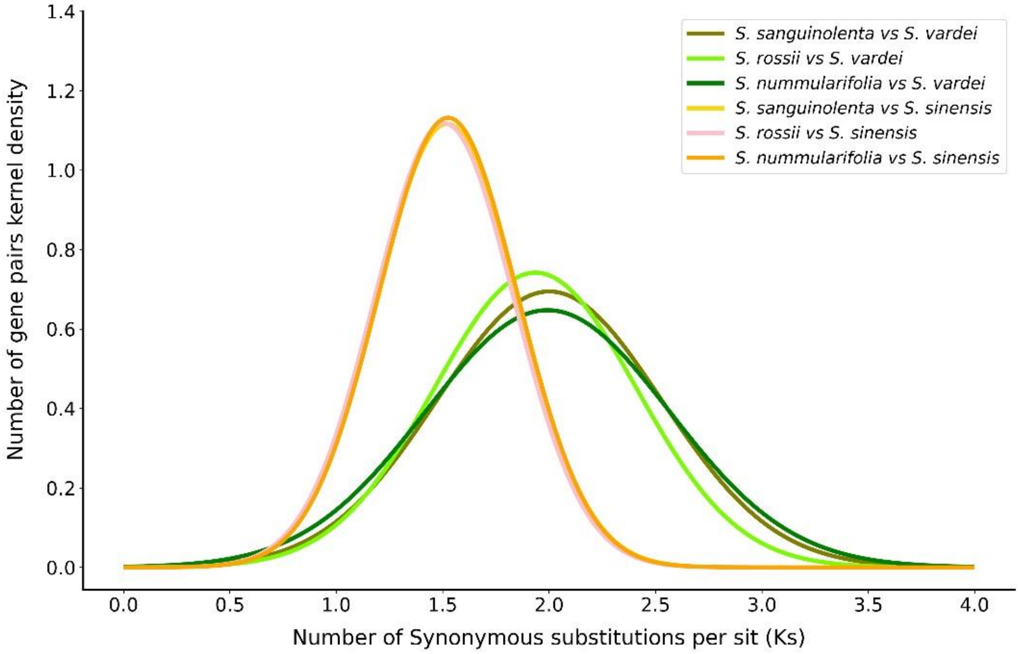

Figure S4. Ks distribution of 347 orthologous genes used for phylogenetic reconstruction. Gaussian fitting of Ks distribution using cftool module in Matlab.

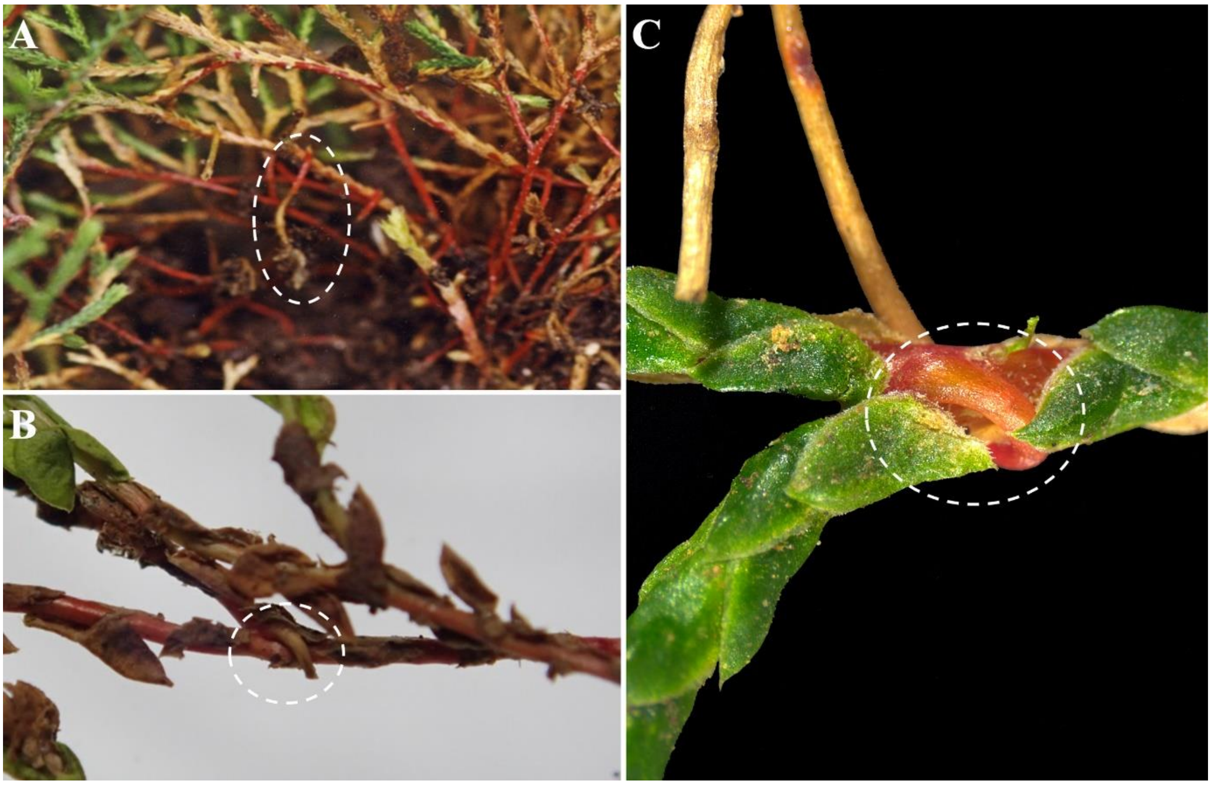

Figure S5. Rhizophores on dorsal side in the *Selaginella sanguinolenta* group. (A and B) Rhizophores of *Selaginella rossii*. (C) Rhizophore of *Selaginella sanguinolenta*. The white dashed circle presents the rhizophore on the dorsal side.

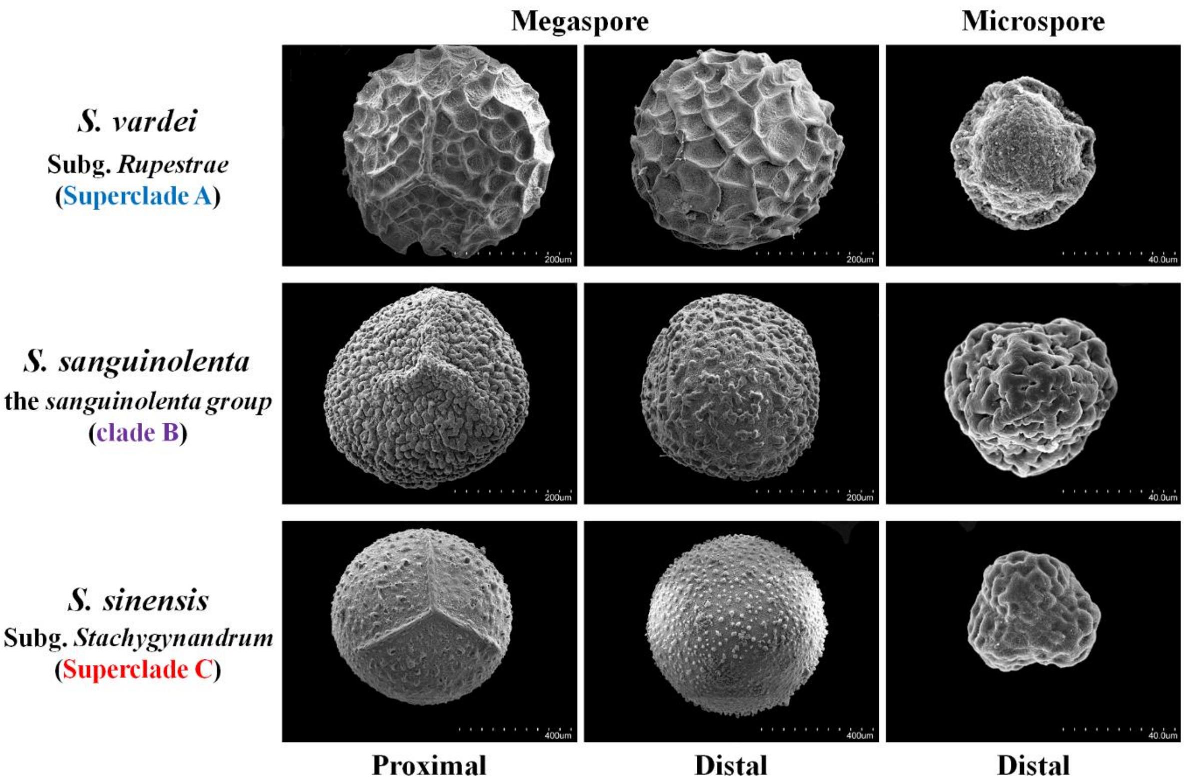

Figure S6. Megaspores and microspores of three representative *Selaginella* species. *S. vardei* belongs to subgenus *Rupestrae* (superclade A), *S. sanguinolenta* represents the *S. sanguinolenta* group, and *S. sinensis* belongs to subgenus *Stachygynandrum* (superclade C).

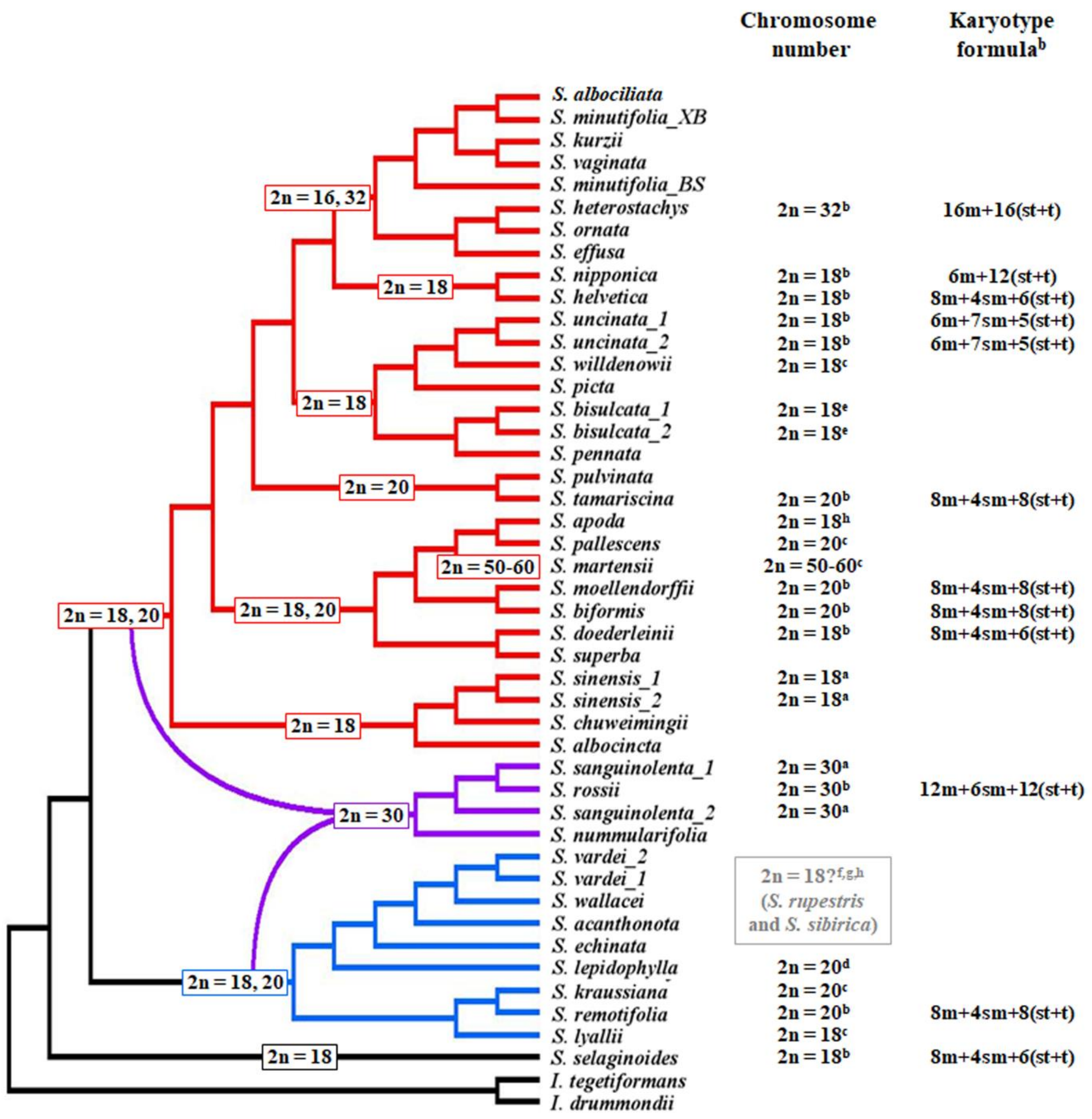

Figure S7. Chromosome evolution of the Selaginellaceae. A species network inferred from nuclear single-gene trees was used as the backbone. The putative ancestral chromosome numbers of each clade were presented on the branches. The observed chromosome numbers and karyotypes of each species from this study and previous studies were presented on the right side of the species network. a: This study, b: Takamiya (1993), c: Jermy et al. (1967), d: VanBuren et al. (2018), e: Mukhopadhyay and Goswami (1996), f: Zhukova and Petrovsky (1975), g: Love and Love (1975), h: Love and Love (1976).

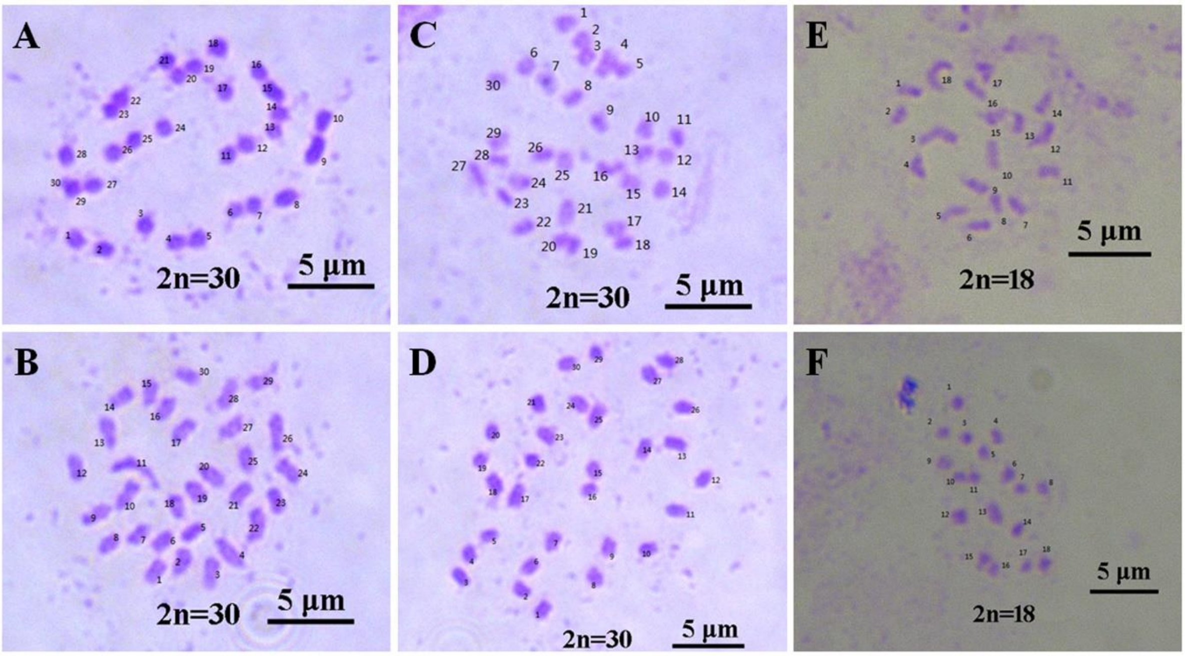

Figure S8. Somatic chromosomes of *Selaginella sanguinolenta* and *S. sinensis*. A-D: Somatic chromosomes at metaphase in *S. sanguinolenta*. E and F: Somatic chromosomes at metaphase in *S. sinensis*.

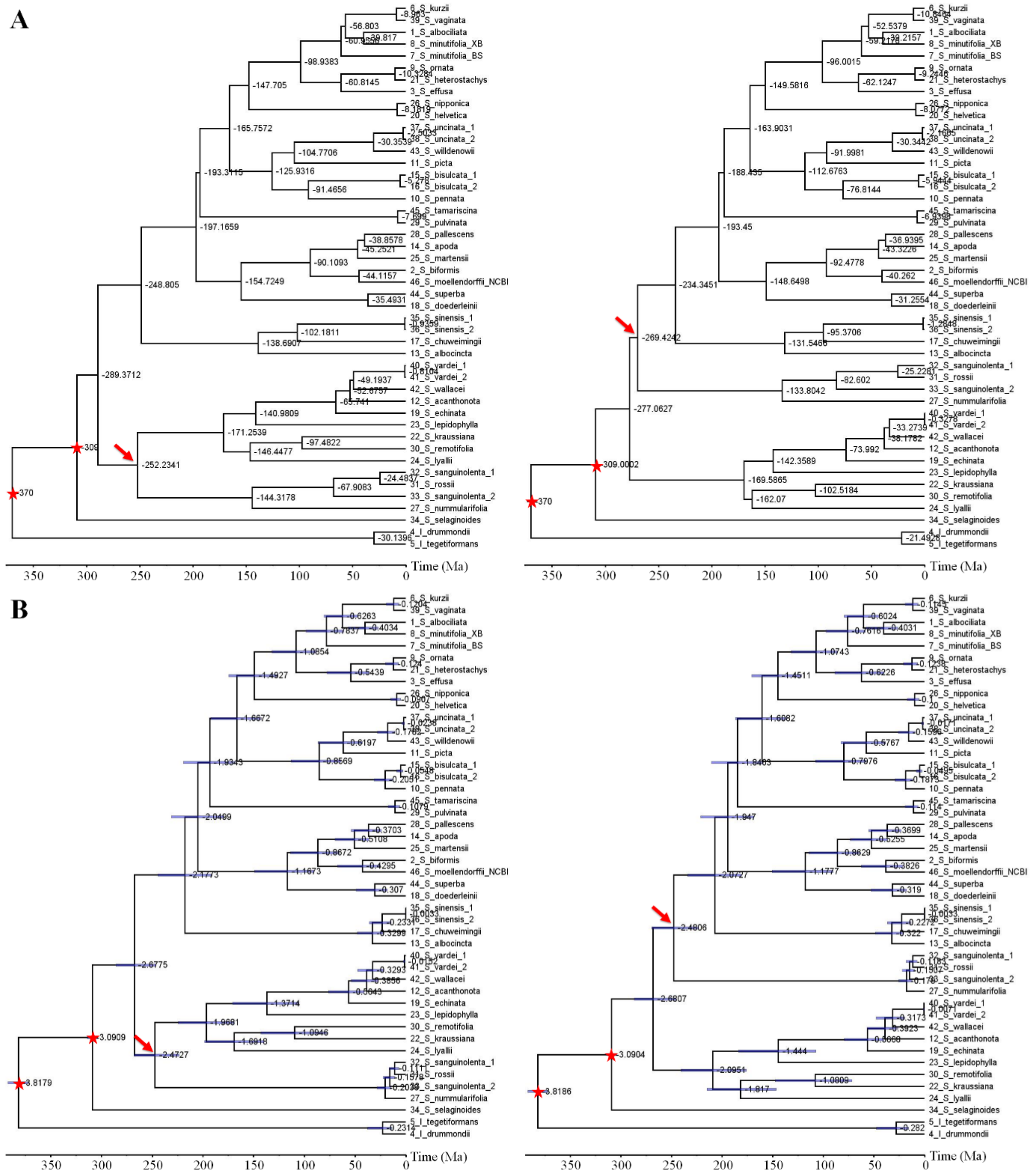

Figure S9. Divergence time estimations for the Selaginellaceae using two different methods. The red stars indicate fossil calibration points. Divergence times were estimated using gene set B (left) and gene set C (right). Red arrows indicate the split between the *S. sanguinolenta* group and superclade A or C, respectively. (A) Divergence time estimated by Penalized likelihood (PL) method. (B) Divergence time estimated by MCMCtree method.

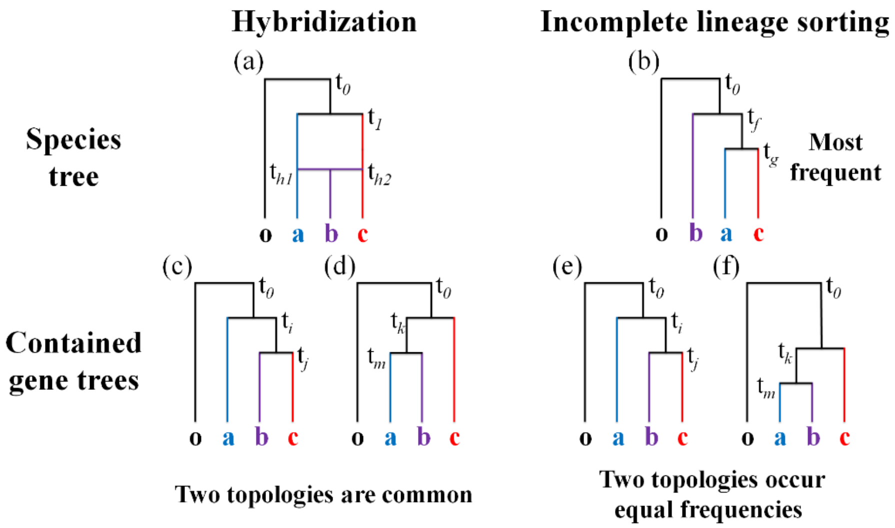

Figure S10. Conceptual differences between hybridization and incomplete lineage sorting. Both concepts of hybridization and incomplete lineage sorting were adopted from Sang and Zhong (2000) and Pamilo and Nei (1988). Representative species trees, where "a", "b", and "c" are ingroup species and "o" is an outgroup species. t_0 indicates the speciation time between the ingroup and outgroup. t_f, t_g, t_i, t_j, t_k, and t_m indicate divergence times, and t_{h1} and t_{h2} indicate the hybridization time giving rise to "b" diverged from "a" and "c". Representative species trees are given in (a, b). The two different gene trees are given in (c, d, e, and f). Expected gene-wise frequencies in the genome are shown near trees (b–f).

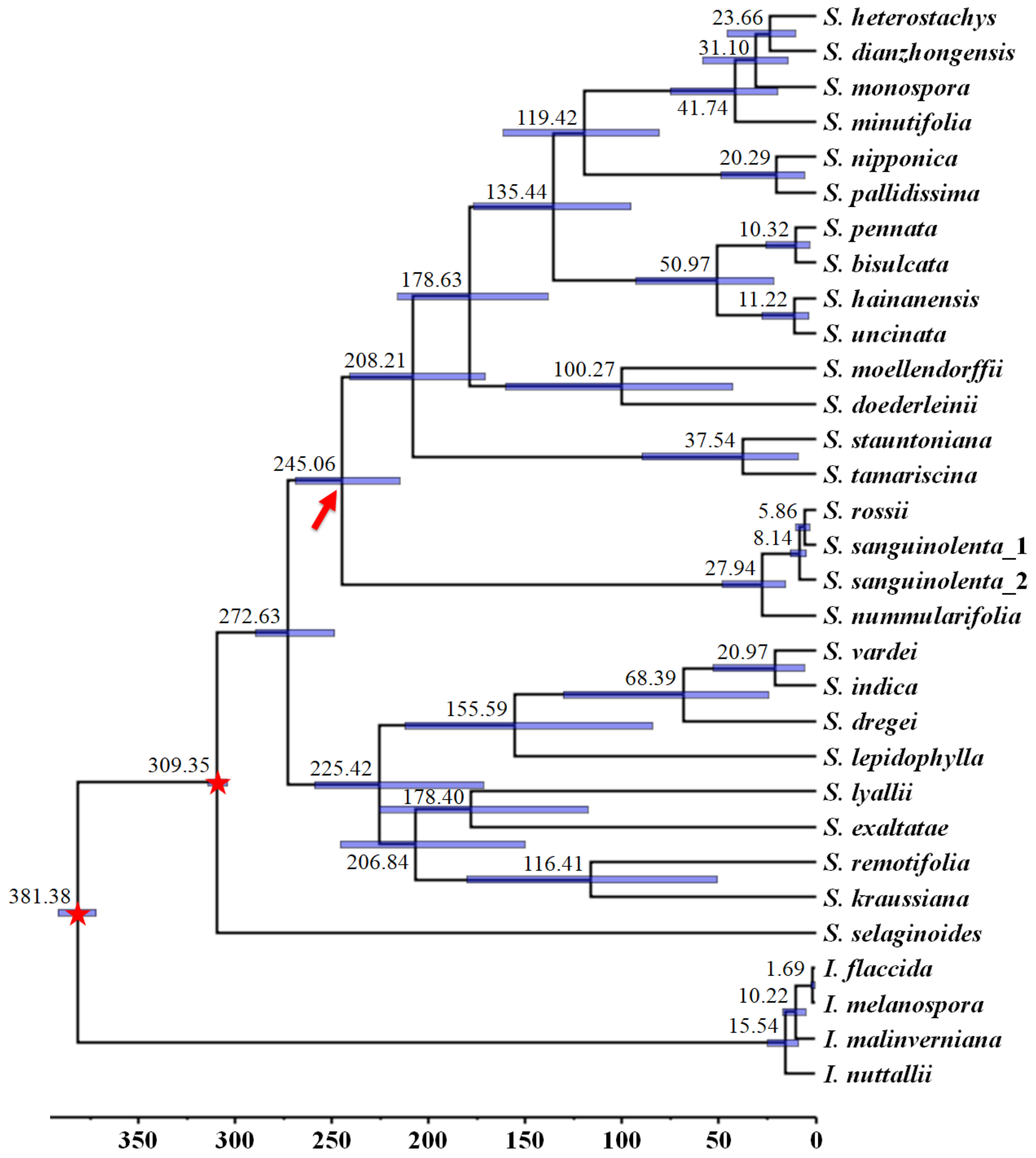

Figure S11. Divergence time estimation for the Selaginellaceae using plastid genome data. The red stars indicate fossil calibration points. Divergence time was estimated by MCMCtree method based on plastid genome data (Zhang et al., 2020). Red arrow indicates the split between the *S. sanguinolenta* group and superclade C

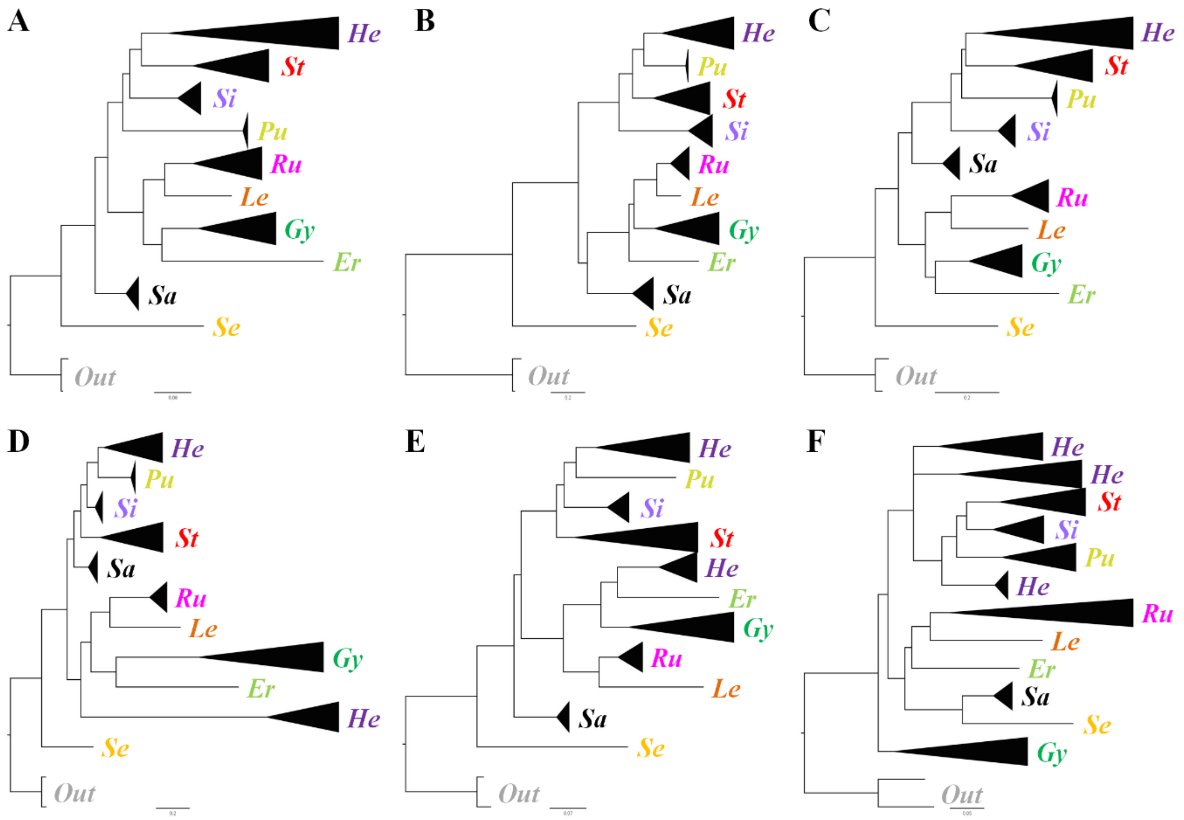

Figure S12. Examples of selected and excluded single gene trees in this study. *Out*: outgroup, *Se*: subg. *Selaginella*, *Er*: subg. *Ericetorum*, *Gy*: subg. *Gymnogynum*, *Le*: subg. *Lepidophyllae*, *Ru*: subg. *Rupestrae*, *St*: the *Stachygynandrum* clade, *He*: the *Heterostachys* clade, *Pu*: *S. pulvinata* clade, *Sa*: *S. sanguinolenta* clade, *Si*: *S. sinensis* clade. *He*, *Pu*, *Sa*, and *Si* belong to subg. *Stachygynandrum sensu* Weststrand and Korall (2016a). The single gene trees showing monophyletic for each lineage were selected for further analyses (A-C). The single gene trees showing nonmonophyletic in some lineages were excluded (D-F).
